# Modes of Information Flow

**DOI:** 10.3390/e28101162

**Published:** 2026-10-21

**Authors:** Ryan G. James, Blanca Daniella Masante Ayala, Bahti Zakirov, James P. Crutchfield

**Affiliations:** 1 Complexity Sciences Center and Physics Department, University of California at Davis, One Shields Avenue, Davis, CA 95616, USA; masante@ucdavis.edu; 2 Institute of Science and Technology Austria, Maria Gugging, 3400 Klosterneuburg, Austria

**Keywords:** stochastic process, transfer entropy, intrinsic mutual information, mutual information, information flow, cryptography, 05.45.-a, 89.70.+c, 05.45.Tp, 02.50.Ey, 02.50.-r, 02.50.Ga

## Abstract

Information flow between components of a system takes many forms and is key to understanding the organization and functioning of large-scale, complex systems. We demonstrate three modalities of information flow from time series *X* to time series *Y*. *Intrinsic* information flow exists when the past of *X* is individually predictive of the present of *Y*, independent of *Y*’s past; this is most commonly considered information flow. *Shared* information flow exists when *X*’s past is predictive of *Y*’s present in the same manner as *Y*’s past; this occurs due to synchronization or common driving, for example. Finally, *synergistic* information flow occurs when neither *X*’s nor *Y*’s pasts are predictive of *Y*’s present on their own, but taken together they are. The two most broadly-employed information-theoretic methods of quantifying information flow—time-delayed mutual information and transfer entropy—are both sensitive to a pair of these modalities: time-delayed mutual information to both intrinsic and shared flow, and transfer entropy to both intrinsic and synergistic flow. To quantify each mode individually we introduce our *cryptographic flow ansatz*, positing that intrinsic flow is synonymous with secret key agreement between *X* and *Y*. Since the secret key agreement rate is not directly computable, we estimate intrinsic flow by an easily-computed upper bound on it—the intrinsic mutual information—so that the resulting three-way split is relative to this bound. We use it to quantify the three flow modalities in a variety of systems including asymmetric flows, coupled chaotic dynamical systems, leader-follower collective dynamics, and financial markets.

## 1. Introduction

Information flow is an important signature of truly complex systems—an incisive proxy for their structure and behavior. Truly complex systems are becoming increasingly familiar to most all the sciences and engineering. Certain expressed genes modulate the expression of others, leading to structured biological processes in metabolism, development, and evolution. The price of a good signals scarcity and demand to consumers and producers, respectively. During specific cognitive tasks, only a small portion of the brain’s connectome is activated and so determining the underlying subnetwork is critical to identifying neural function. To these ends, methods of tracking and quantifying information flow form a core toolset for analyzing large-scale complex systems.

Methodologically, it is difficult to find an alternative toolset with the many features that recommend using information theory [1,2] to analyze complex systems. First, information accounts for any type of co-relation [1]. Whereas, statistical correlation requires an underlying model and typically captures only linear statistical dependencies [3]. Second, information is broadly applicable. Many systems across the sciences simply do not have an “energy” and this precludes appealing to physics-based modeling that starts from a system Hamiltonian. In contrast, information can be defined for mechanical, chemical, biological, social, and engineered systems. Third, information provides directly comparable quantitative units across qualitatively different systems. This is unlike pairwise statistical correlation which comes in fixed, domain-specific units—meters, concentration, volts, and dollars squared, and so on—while information is universally measured in bits. Fourth, there is a rough equivalence between probability theory and information theory [4] and a substantial foundational overlap between mathematical statistics and information theory; e.g., see Refs. [1,5].

Finally, and perhaps most importantly, large-scale complex systems generate emergent patterns—patterns that an analyst does not know a priori. Information does not require prior knowledge of an appropriate representational basis, which is essential when attempting to discover new patterns not seen before [6]. Despite a number of technical challenges, to date it appears that the tools of information are the most general, workable alternative available in the pursuit of complex systems. The following provides new results that remove several of the remaining impediments to its even broader use.

In particular, despite its long-lived intuitive appeal [7,8,9,10,11], information flow is not yet a concretely defined concept. One consequence is that many methods of calculating it fail surprisingly when deployed in unfamiliar contexts [12]. Here, we posit that these failures are not directly due to shortcomings of the quantitative measures themselves, but rather arise due to analysts adhering to the concept of a unitary information flow. In contrast, we demonstrate that the flow between two time-series, $X=\ldots ,X_{t},X_{t+1},\ldots$ and $Y=\ldots ,Y_{t},Y_{t+1},\ldots$, takes on three qualitatively distinct modes: *intrinsic*, *shared*, and *synergistic* information flows. Unfortunately, to date methods of computing information flow do not quantify these distinct modes. Here, we solve this problem via a novel adaptation of cryptography.

Our development proceeds as follows. Section 2 discusses and exemplifies the three modes of information flow. As an aid in this, Appendix A briefly reviews the necessary notation and concepts from elementary probability (random variables *X* and *Y*), time series (random variable sequences $X_{t:t^{\prime }}$), and information measures ($H\left[X\right],H\left[Y|X\right],I\left[X:Y\right],I\left[X:Y|Z\right]$). Section 3 then surveys extant measures of information flow within multivariate time series. Section 4 introduces our *cryptographic flow ansatz* and shows how it isolates intrinsic information flow and so yields the full three-way decomposition of information flow. Section 5 explores components of the decomposition in a variety of settings, including asymmetric flows, coupled chaotic dynamical systems, leader-follower dynamics in agent collectives, and financial indices.

An earlier version of this work was circulated as a preprint in 2018 [13]. Several studies have since applied or extended the framework: to collective motion [14], to neural estimation of the intrinsic flow [15], and to its use in hypothesis testing [16]. Although some of these appeared in print first, they build on the framework presented here; we discuss them in Section 5.3 and Section 5.5.

A series of appendices rounds-out the main development, giving background on information in time series and multivariate systems, differences with the partial information decomposition, computational methods for information measures, details on financial time series, and the statistical significance of informational estimates.

## 2. Modes of Information Flow

Colloquially, information flow is the movement of information from one agent or system to another. Unlike many flows considered in physics, such as electric current or fluid flow, there is no single conservation law for information. This makes quantifying information flow vastly more challenging. In light of this, definitions of information flow have been somewhat ad-hoc, though they typically employ either some form of mutual information [17] or quantify the influence one agent has on another [18]. They are often backed with examples where the proposed measure performs admirably, though performance in other settings can be mixed or downright misleading. We propose that these inconsistencies are due to conflating functionally distinct modes of information flow.

Specifically, information flow from time series *X* to time series *Y* can take three qualitatively distinct forms. The first, *intrinsic flow*, is when the past behavior of the *X* time series is directly predictive of the present behavior of the *Y* time series in a fashion that the past behavior of *Y* is not. For example, this occurs when an “upstream” *X* drives a “downstream” *Y*. The second mode, *shared flow*, is when the present behavior of the *Y* time series can be inferred from the prior behavior of either the *X* time series or the *Y* time series. This occurs due to, say, a common driver or synchronization within a system. The third mode, *synergistic flow*, occurs when both the past of the *X* time series and the past of the *Y* time series are each independent of the present of the *Y* time series, but when combined the two pasts become predictive of it. This occurs in systems where the behavior of a component strongly depends upon its context within the system. Appendix B gives a more detailed rationale for the three modalities of flow.

**Figure 1:**
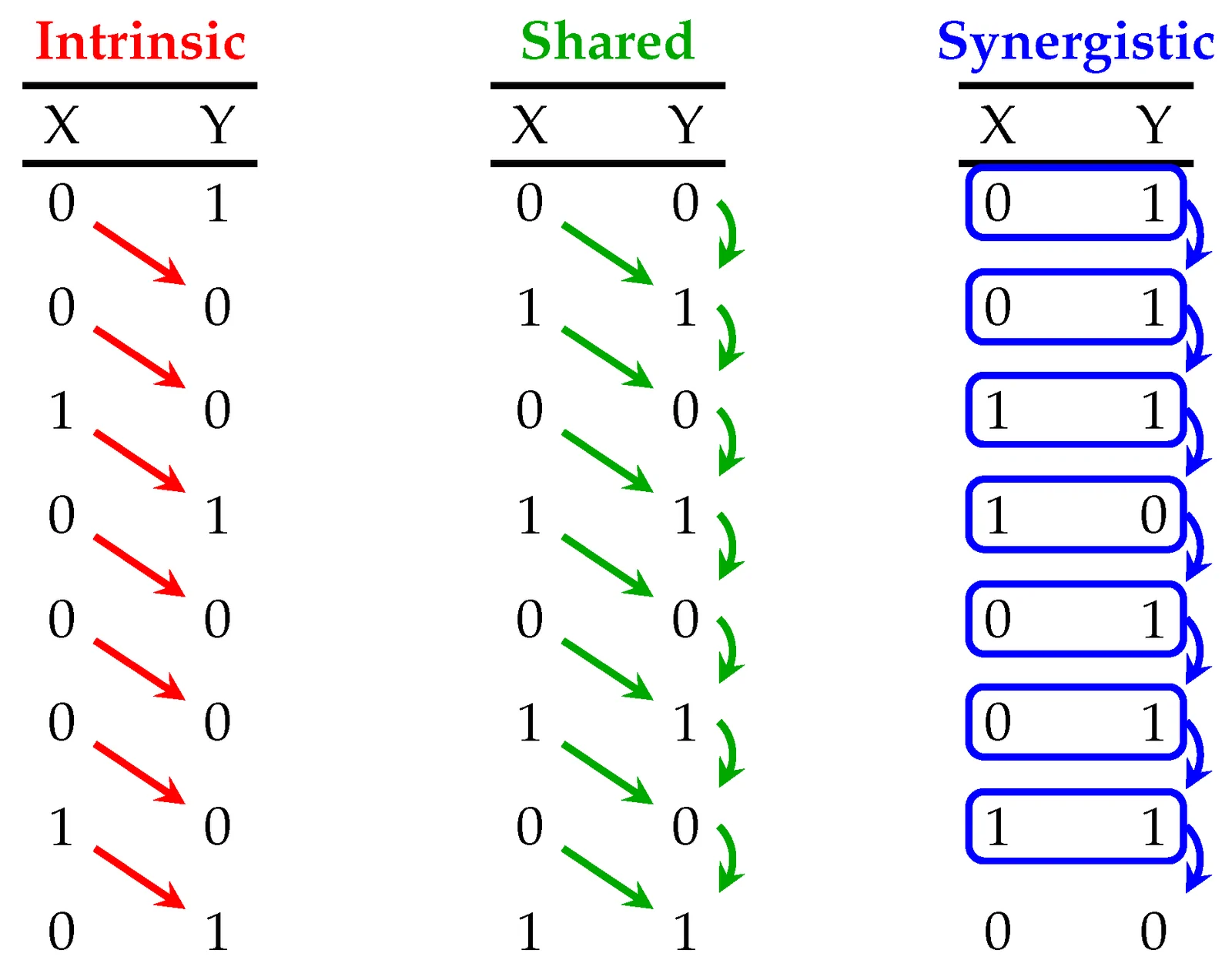
Modes of information flow: Intrinsic flow is exemplified by $Y_{0}=X_{-1}$. Shared flow by $Y_{0}=\neg X_{-1}=\neg Y_{-1}$. Synergistic flow by $Y_{0}=X_{-1}⊕Y_{-1}$.

Markovian examples of these three types of flow are illustrated in Figure 1. Exemplifying intrinsic flow is the case where $X_{t}$ is random and *Y* simply follows it: $Y_{0}=X_{-1}$. Shared flow is demonstrated with synchronization, where $Y_{0}=\neg X_{-1}=\neg Y_{-1}$, where ¬ is the “not” operation. Finally, synergistic flow can be seen when $X_{t}$ is random and $Y_{0}=X_{-1}⊕Y_{-1}$, where ⊕ is the exclusive-OR operation. Both shared and synergistic flows are symmetric, in that they cannot be said to originate from either *X*’s past or *Y*’s past. Whereas, intrinsic flow is uniquely attributed to *X*’s past.

Now, the question is: How do we detect and quantify these three forms of dependence from time series observations?

## 3. Extant Measures of Information Flow

Historically, information flow has been measured via the *time-delayed mutual information* [19]:

(1) $$\begin{array}{c} I\left[X_{-1}\;:\;Y_{0}\right]\;. \end{array}$$

**Figure 2:**
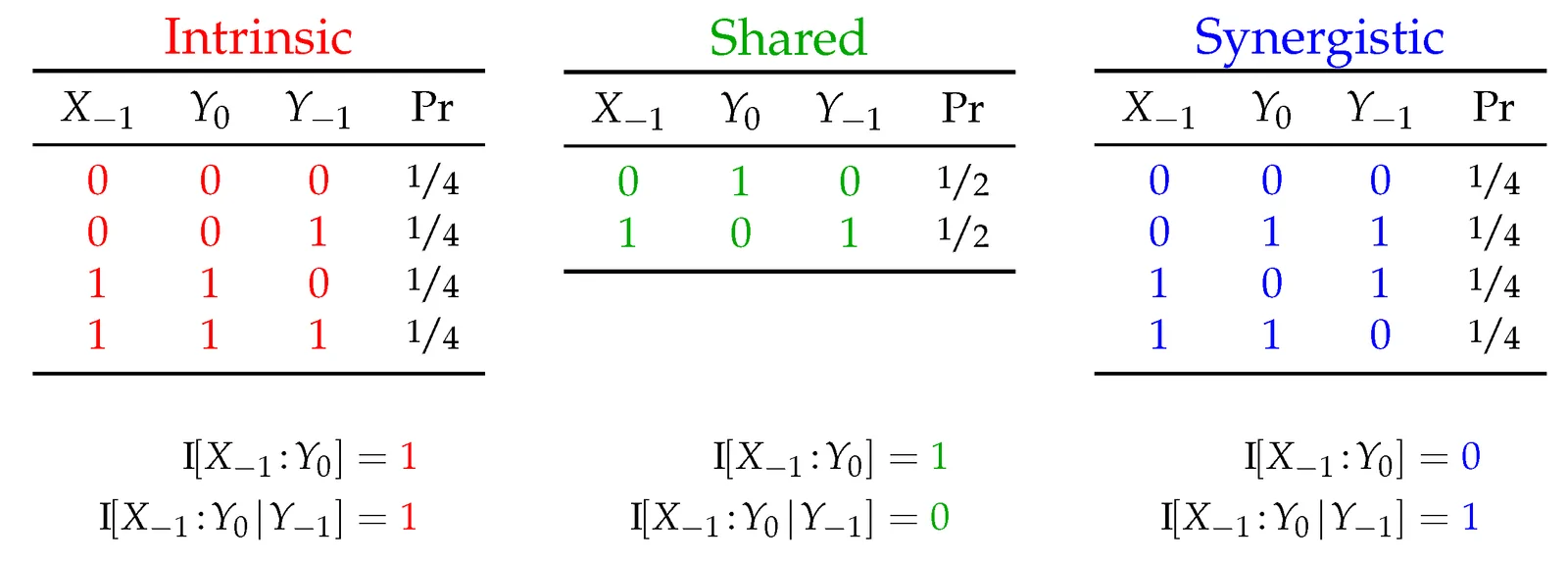
Three canonical types of dependence between two variables $X_{-1}$ and $Y_{0}$ in the context of a third $Y_{-1}$: (**Left**) Intrinsic dependence exists between the first two variables in spite of the third. (**Middle**) Shared dependence exists synchronously with the third. (**Right**) Synergistic dependence exists only when also observing the third.

It posits that information flow from *X* to *Y* is the information shared between *X*’s past observations $X_{-1}$ and *Y*’s present observation $Y_{0}$. As such, it is sensitive to both intrinsic and shared dependence, as seen in Figure 2. While time-delayed mutual information captures a restricted notion of causality, it “…fails to distinguish information that is actually exchanged from shared information due to common history and input signals” [20]. That is, it conflates intrinsic and shared dependence.

To cleave away the shared dependence from the time-delayed mutual information Ref. [20] proposed the *transfer entropy*:

(2) $$\begin{array}{c} I\left[X_{-1}\;:\;Y_{0}\;\;|\;\;Y_{-1}\right]\;, \end{array}$$

—the information shared by *X*’s past and *Y*’s present, given *Y*’s past—and correctly intuited that the influences of common history and input signals on shared information “…  are excluded by appropriate conditioning of transition probabilities”.

Unfortunately, this ignores the possibility of conditional dependence. And, the transfer entropy suffers as a result: It fails to distinguish intrinsic flow from synergistic flow; again, see Figure 2. (That transfer entropy separates into two components is not new, and the integrated information decomposition [21] resolves both it and the time-delayed mutual information into finer atoms; see Appendix C). Taken at face value, this presents the unfortunate situation of being unable to quantify any specific mode of information flow.

A short aside will highlight the issue here. Previous efforts to measure flow rest on the misunderstanding that conditioning is *subtractive*: “in our new approach, these influences are excluded by appropriate conditioning of transition probabilities” [20]. The erroneous assumption here being that conditioning only *excludes* dependency. In point of fact, information-theoretic conditioning and probabilistic conditioning, for that matter, are generically *not* subtractive operations, as demonstrated in Figure 2 and Problem 6 in Chapter 2 of Ref. [1]. Ignoring this has led to the belief that conditioning on more and more time series results in a more incisive analysis of information flow within a system [20,22,23]. It need not.

## 4. Cryptographic Common Information

Overcoming the challenge of information flow requires adopting a different viewpoint—one that directly addresses when two system components, and only two system components, possess common information. Solving this would circumvent the open-ended issue of conditioning on all of a system’s other, possibly unspecified, variables besides the two of interest; a strategy that, on its own, fatally ignores conditional dependence, as we just argued. Thus, we must simultaneously solve a definitional problem and a technical problem: respectively (i) acknowledging distinct modes of flow and (ii) accounting for both conditional independence and conditional dependence. Our solution appeals to cryptography and the information theory of two parties sharing secret keys. We introduce an ansatz that directly quantifies intrinsic flow and, thereby, completes the decomposition of information flow into its three modes.

Consider again the flow of intrinsic information from $X_{-1}$ to $Y_{0}$. The flow implicates some sort of dependency or correlation between *X* and *Y* that can unambiguously be attributed to *X*. Were this dependency able to be reproduced from other aspects of the system, it could not be said to have originated from $X_{-1}$. This observation evokes the cryptographic idea of *secret key agreement* and leads to our ansatz:

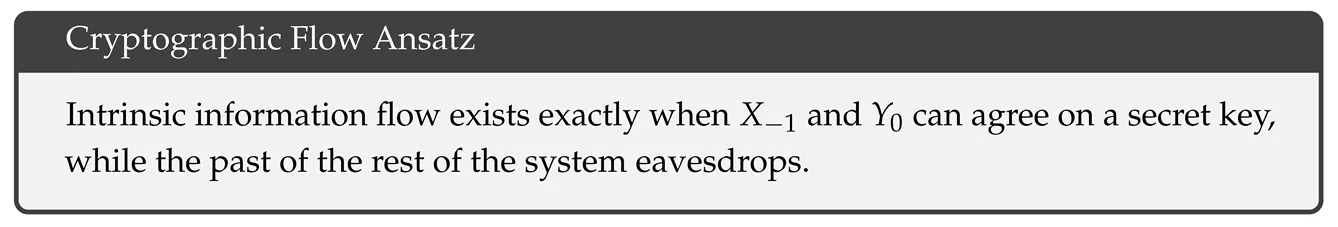



Quantitatively, intrinsic information flow is the rate at which information secret to $X_{-1}$ and $Y_{0}$ can be extracted from observations of the system.

### 4.1. Secret Key Agreement Rate

In this way, we identify the intrinsic information flow as the secret-key agreement rate [24], which is defined as follows. Consider a joint random variable (X,Y,Z), where Alice has access to the *X* realizations, Bob the *Y* realizations, and Eve the *Z* realizations. Given *N* IID realizations of the joint variable, let $X^{N}$ denote Alice’s observations, $Y^{N}$ Bob’s, and $Z^{N}$ Eve’s. Random variable *V* represents the public communication that all three observe. Let *S* denote the secret key that Alice and Bob wish to have in common.

Now, let $S_{X}$ and $S_{Y}$ represent the secret keys that Alice and Bob, respectively, distill from their private observations as well as from the public communication *V*: $S_{X}=f\left(X^{N},V\right)$ and $S_{Y}=g\left(Y^{N},V\right)$. In this, functions *f* and *g* represent the mechanism by which Alice and Bob construct their copy of secret key *S*. A *secret key agreement scheme* defines the allowed public communications *V* and mechanisms *f* and *g*. If the scheme is any good, $S_{X}$ and $S_{Y}$ will be identical and equal to *S* with high probability: $\mathrm{Pr}(S_{X}=S_{Y}=S)\geq 1-\epsilon$. Moreover, being secret, the key *S* should have arbitrarily small correlation with Eve’s private observations *Z* and the public communication *V*: $I\;\left[S:V,Z^{N}\right]\leq \epsilon$. The *secret-key agreement rate* S(X:Y||Z) then is the maximum rate *R* such that:

$$\begin{array}{c} \lim_{N\to \infty }\frac{1}{N}H\;\left[S\right]\geq R-\epsilon \;, \end{array}$$

for N>0 and ϵ>0. In other words, the secret key agreement rate is the largest rate at which a secret key *S* can be successfully produced.

Effectively, given realizations of the three-way joint random variable, there exists a scheme by which Alice and Bob can publicly exchange information and then distill their public and private information into a secret key upon which they both agree with arbitrarily high probability, but which has arbitrarily little information shared with all information available to Eve. The challenge now is to determine the secret-key extraction functions *f* and *g*, as well as what public communication *V* is necessary. See Ref. [25] for concrete examples.

### 4.2. An Easily Computed Upper Bound

Though we identified the secret key agreement rate with intrinsic information flow, the nonconstructive nature of its definition mandates we appeal to some proxy if we wish to practically estimate it. While several lower and upper bounds exist for the secret key agreement rate, here we use the *intrinsic mutual information* [26]. We recommend this upper bound due to its nontrivial behavior (exemplified shortly) and relatively straightforward estimation.

An eavesdropper not only has access to her observations *z*, but also to any (local) modification $\mathrm{Pr}(\bar{z}|z)$ of them. Therefore, the ability of Alice and Bob to agree upon a key cannot be reduced if *Z* is replaced by any “corruption” $\bar{Z}$. This observation simplifies the secret key optimization, leading to a constructive bound on the secret key agreement rate:

(3) $$\begin{array}{cc} S(X\;:\;Y\;||\;Z) & \leq \min_{\mathrm{Pr}(\bar{z}|z)}I\;\left[X:Y\;|\;\bar{Z}\right] \end{array}$$

(4) $$\begin{array}{cc} \; & =I\;\left[X:Y↓Z\right]\;. \end{array}$$

The last quantity—*intrinsic mutual information*—is therefore an upper bound on the secret key agreement rate. It can be easily verified that $I\;\left[X:Y↓Z\right]$ is bounded from above by both $I\;\left[X:Y\right]$, when $\mathrm{Pr}(\bar{z}|z)$ is constant, and $I\;\left[X:Y\;|\;Z\right]$, when $\mathrm{Pr}(\bar{z}|z)$ is the identity.

Fortunately, this optimization is not difficult due to the boundedness of $\bar{Z}$. (In fact, $|\bar{Z}\left|\;\leq \;|Z\right|$ [27]). Appendix D explains how to calculate the intrinsic mutual information and also addresses three practical concerns.

First, the intrinsic mutual information is only one of several known bounds on S(X:Y||Z), and the gap can be qualitative. Gisin and Wolf conjectured the existence of *bound information*, distributions with positive intrinsic mutual information but a vanishing secret key agreement rate [28]. Pauwels et al. recently exhibited one, consisting of two bits and a trit [29]. Our intrinsic flow, and with it the three-way split, is therefore relative to the chosen bound. Where tighter limits on the flow rates are needed, the upper bounds (the reduced, minimal, less-noisy, and two-part intrinsic mutual informations) and lower bounds (from no, one-way, and interactive communication) surveyed in Appendix D.1 can be leveraged to bracket the secret key agreement rate more closely. Every one of these lies below $\min\{I\;\left[X_{-1}\;:\;Y_{0}\right],I\;\left[X_{-1}\;:\;Y_{0}\;\;|\;\;Y_{-1}\right]\}$ (the time-delayed mutual information and the transfer entropy), so substituting any of them for the intrinsic mutual information keeps the flow decomposition of Section 4.3 nonnegative. The lower bounds, moreover, certify that a secret key can be agreed, and so identify at least some cases in which intrinsic flow is strictly nonzero, whichever upper bound is used to quantify it. For the flow from *Y* to *X* in Section 5.1, for example, the one-way lower bound $I\;\left[Y_{-1}:X_{0}\right]-I\;\left[Y_{-1}:X_{-1}\right]$ equals the intrinsic mutual information, 0.526200 bit, so there the intrinsic flow is exactly the secret key agreement rate.

Second, computing the intrinsic mutual information directly for continuous-valued sources is fraught (Appendix D.2). One can instead leverage discretization, applying the discrete method to binned, ordinal, or generating-partition symbolizations of the signals, subject to the caveats of Appendix D.3.

Finally, the intrinsic mutual information may itself be directly a measure of interest, independent of its role as a bound on S(X:Y||Z). In the flow setting, $I\;\left[X_{-1}:Y_{0}↓Y_{-1}\right]$ is the amount of information that the past of *X* and the present of *Y* share given *any* transformation of *Y*’s past—the dependence that no processing of the target’s own history can explain away.

Throughout, we take the eavesdropper to be the past of the target alone, so that intrinsic flow is $I\;\left[X_{-1}:Y_{0}↓Y_{-1}\right]$. In a system of more than two components, the eavesdropper should strictly hold the pasts of all components other than the source, so that dependence relayed through, or driven by, third components is not credited to the source. We adopt the target’s past only as an approximation and simplification, as is common in pairwise analyses of information flow, which likewise condition the transfer entropy on the target’s own past rather than on the full remainder of the system [23]. Conditioning on the remaining components instead, as causation entropy does for the transfer entropy [22], would give the eavesdropper more information, which can only lower the intrinsic flow. For the two-component systems of Section 5.1 and Section 5.2 the two choices coincide; for the financial application of Section 5.4, where the other constituent stocks are omitted, they do not.

**Figure 3:**
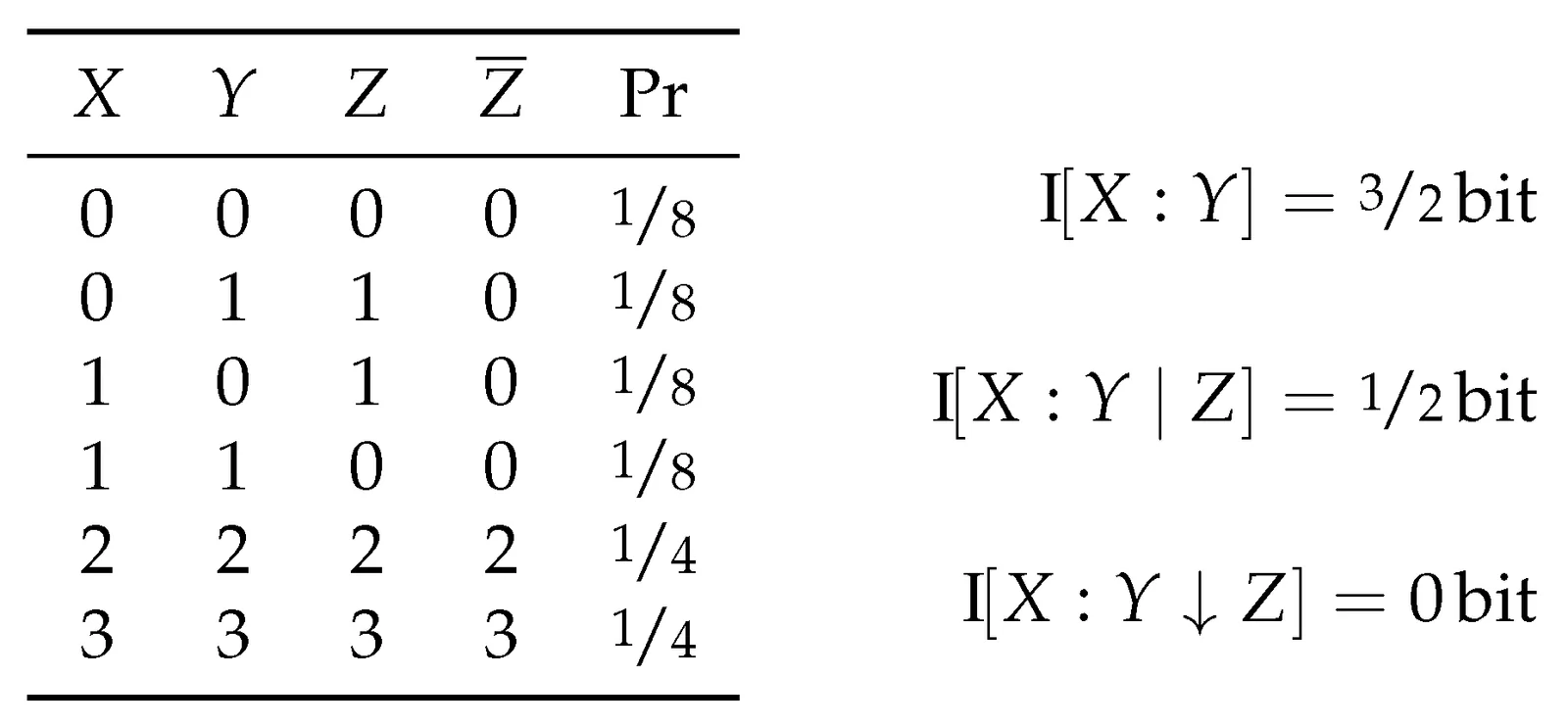
The intrinsic mutual information $I\;\left[X:Y↓Z\right]$ can be less than both $I\;\left[X:Y\right]$ and $I\;\left[X:Y\;|\;Z\right]$: The mutual information $I\;\left[X:Y\right]$ captures the 01-, 2-, and 3-ness that is shared by *X* and *Y*, while the conditional mutual information $I\;\left[X:Y\;|\;Z\right]$ captures the fact that knowledge of *Z* indicates whether the 0s and 1s of *X* and *Y* are the same or different. Neither of these dependencies are held by *X* and *Y* alone, and so the intrinsic mutual information $I\;\left[X:Y↓Z\right]$ vanishes.

To illustrate its behavior, consider the distribution in Figure 3 [26]. This distribution has two qualitatively distinct sets of events. The first, encoded using 0s and 1s, exhibits conditional dependence: any pair of *X*, *Y*, or *Z* are independent, but given the third they are perfectly correlated. The second, encoded using 2 s and 3 s, exhibits conditional independence: any pair is perfectly correlated and is also correlated with the third. The mutual information IX:Y=H12,14,14=32 bit, reflecting that they share 01-, 2-, and 3-ness. However, the conditional mutual information IX:Y|Z=12 bit, reflecting the conditional dependence that occurs half the time. The mapping of *Z* to $\bar{Z}$ given in the table demonstrates that the conditional dependence can be destroyed while preserving the conditional independence: $I\;\left[X:Y\;|\;\bar{Z}\right]=0\;\mathrm{bit}=I\;\left[X:Y↓Z\right]$. This indicates that both the mutual information and conditional mutual information misleadingly identify dependencies that do not belong to *X* and *Y* alone, but rather are shared by, or induced by, *Z*.

### 4.3. Flow Decomposition

Recall that we showed the time-delayed mutual information $I\;\left[X_{-1}\;:\;Y_{0}\right]$ captures both intrinsic and shared flows, while transfer entropy $I\;\left[X_{-1}\;:\;Y_{0}\;\;|\;\;Y_{-1}\right]$ captures intrinsic and synergistic flows. Together with our cryptographic flow ansatz that quantifies intrinsic flow, simple algebra now gives a full and constructive decomposition of the distinct flow modes:

**ntrinsic Flow** $I\;\left[X_{-1}\;:\;Y_{0}↓Y_{-1}\right]$
**Shared Flow** $I\;\left[X_{-1}\;:\;Y_{0}\right]\;-\;I\;\left[X_{-1}\;:\;Y_{0}↓Y_{-1}\right]$
**Synergistic Flow** $I\;\left[X_{-1}\;:\;Y_{0}\;\;|\;\;Y_{-1}\right]\;-\;I\;\left[X_{-1}\;:\;Y_{0}↓Y_{-1}\right]$

Since the intrinsic mutual information is bounded from above by both the mutual information and the conditional mutual information (Section 4.2), $I\;\left[X_{-1}\;:\;Y_{0}↓Y_{-1}\right]\leq \min\left\{I\;\left[X_{-1}\;:\;Y_{0}\right],I\;\left[X_{-1}\;:\;Y_{0}\;\;|\;\;Y_{-1}\right]\right\}$. The shared and synergistic flows are therefore automatically nonnegative.

**Figure 4:**
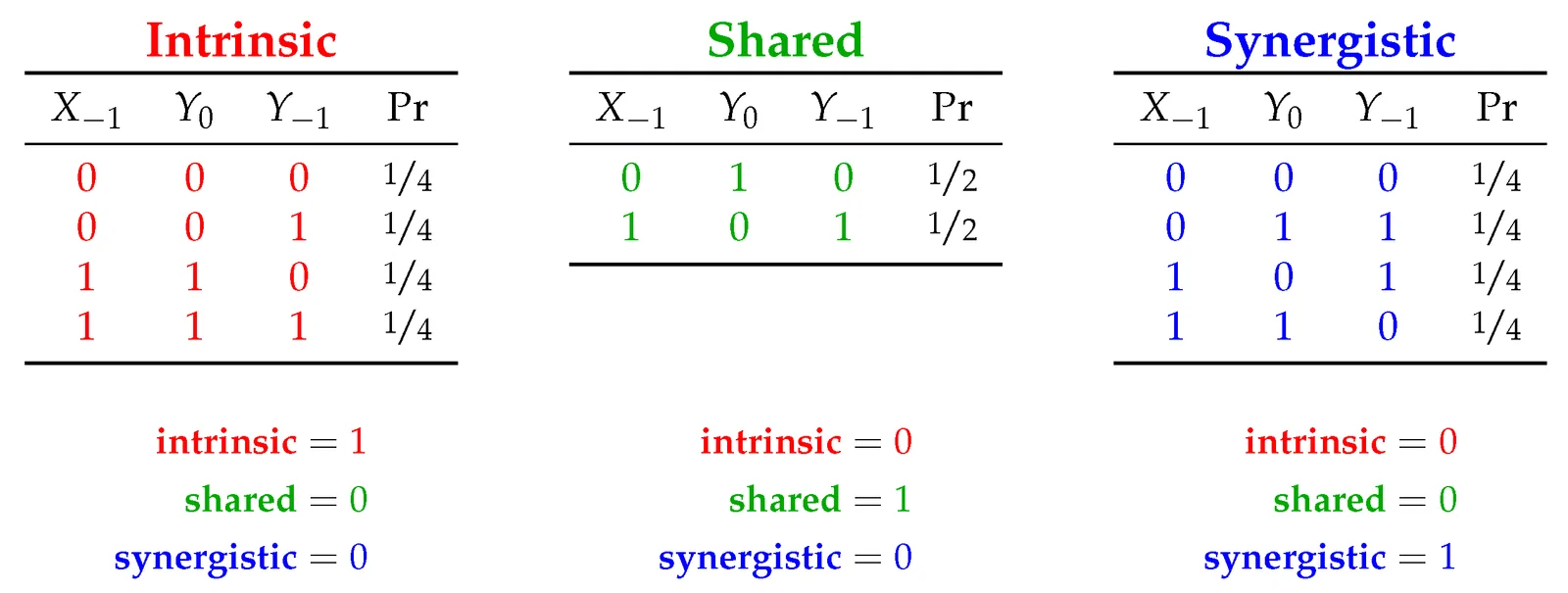
Flow decomposition of the canonical dependency types of Figure 2: Each dependency type is associated with a unique flow mode: intrinsic with intrinsic, shared with shared, and synergistic with synergistic.

To illustrate how this works, Figure 4 returns to the canonical dependency types of Section 2 and applies the decomposition to each. In the case of the Intrinsic pair of time series, we find that intrinsic flow is 1.0 bit while shared and synergistic are both 0.0 bit. The synchronized or Shared pair of time series decomposes with 1.0 bit of shared flow, with 0.0 bit of both intrinsic and synergistic flows. Finally, the Synergistic pair has no intrinsic or shared information flow, but 1.0 bit of synergistic flow. These observations justify the names given to the three modes.

The three modes do not, however, partition any fixed quantity. Their sum,

$$\begin{array}{c} I\;\left[X_{-1}\;:\;Y_{0}\right]+I\;\left[X_{-1}\;:\;Y_{0}\;\;|\;\;Y_{-1}\right]-I\;\left[X_{-1}\;:\;Y_{0}↓Y_{-1}\right]\;, \end{array}$$

lies between the larger of the time-delayed mutual information and the transfer entropy and their sum. In general it equals none of these, nor the joint information $I\;\left[X_{-1},Y_{-1}:Y_{0}\right]$ that a partial information decomposition would apportion (Appendix C). In the information diagram of Appendix B, it is the time-delayed mutual information less the co-information of $X_{-1}$, $Y_{0}$, and $Y_{-1}$ conditioned on the optimal corruption $\bar{Y_{-1}}$. We therefore do not constrain the flows to any particular sum, in line with the absence of any conservation law governing information [12].

### 4.4. Mechanisms of Synergistic Flow

The XOR of Figure 4, in which the effect of $X_{-1}$ on $Y_{0}$ reverses sign with $Y_{-1}$, is the canonical source of synergistic flow. More generally, synergistic flow arises whenever conditioning on the target’s past *creates* dependence between $X_{-1}$ and $Y_{0}$ that no corruption of $Y_{-1}$ preserves. Other mechanisms do this too. In *gating*, $Y_{-1}$ determines whether $X_{-1}$ reaches $Y_{0}$ at all. In *saturation*, the target responds to the source only in some of its own states, as in $Y_{0}=X_{-1}\wedge Y_{-1}$ ( 0.189 bit of synergistic flow when $X_{-1}$ and $Y_{-1}$ are independent uniform bits); this is generic for dynamics with thresholds or folds. In *masking*, the target’s own variability hides the source until it is conditioned away. Even the purely additive $Y_{0}=X_{-1}+Y_{-1}$, with $Y_{-1}$ uniform over *k* values, has 1−1k bit of synergistic flow, as do linearly coupled Gaussian processes [30], so synergy is not by itself a signature of nonlinear interaction. Nor need it reflect a mechanism at all: if $Y_{-1}$ is a common effect of $X_{-1}$ and a hidden driver of $Y_{0}$, conditioning on it induces dependence between its causes, and the XOR distribution can arise even though $X_{-1}$ has no influence on $Y_{0}$.

*Exclusion constraints* are a further mechanism, exemplified by the W distribution [31]: $\left(X_{-1},Y_{0},Y_{-1}\right)$ uniform over 100, 010, and 001, so that exactly one bit is set, as with a single token or one-hot encoding. When $Y_{-1}=1$ the token is accounted for and $X_{-1}=Y_{0}=0$, closing the channel. When $Y_{-1}=0$, $Y_{0}=\neg X_{-1}$ and one bit passes perfectly. Hence IX−1:Y0|Y−1=23 bit, while marginally the two contexts pool into a weak anticorrelation, IX−1:Y0=log23−43≈0.252 bit. The optimal corruption discards $Y_{-1}$ entirely, so the flow is 0.252 bit intrinsic, 0 shared, and $2-\log_{2}3\approx 0.415$ dit synergistic. Unlike the XOR, the W distribution’s synergy coexists with intrinsic flow, and its shared flow vanishes even though $X_{-1}$ and $Y_{-1}$ are dependent.

## 5. Applications

With their definitions and estimation methods laid out, we now turn to demonstrate the diversity and advantage of quantifying separate modes of information flow in settings ranging from asymmetric flow, coupled chaotic dynamical systems, and leader-follower group dynamics to financial indices. We do end, though, with a cautionary exploration of biases that arise in estimating nonnegative statistics, such as the information measures here.

### 5.1. Asymmetric Information Flow?

At first blush, it seems difficult for there to be shared information flow from *X* to *Y*, but not from *Y* to *X*. Here, we provide a relatively simple example and an intuitive explanation of this phenomenon.

Consider the following (jointly) Markovian transition matrix *T* defining a pair of time series *X* and *Y*:

(5) TX−1,Y−1→X0,Y0= 0,00,11,00,00,11,0(01000149590)

with stationary distribution π=211,922,922. The time series’ temporal joint distribution is:
$X_{-1}$$Y_{-1}$$X_{0}$$Y_{0}$Pr0001211011092210002111001522

The joint time series has information flows from *Y* to *X* of 0.526200 bit intrinsic, 0.449821 bit shared, and 0.0 bit synergistic; and from *X* to *Y* of 0.0 bit intrinsic, 0.044381 bit shared, and 0.120759 bit synergistic. Note that from *Y* to *X*, the intrinsic information flow is equal to the transfer entropy and the time-delayed mutual information splits nontrivially into intrinsic and shared flow. From *X* to *Y*, the intrinsic flow vanishes, so the shared flow equals the time-delayed mutual information and the synergistic flow equals the transfer entropy.

To gain a more intuitive understanding of the asymmetry in this, let us isolate the transition matrix corresponding to the generation of the *X* time series:

(6) $$T[X_{-1},Y_{-1}\to X_{0}]=\begin{array}{cc} \begin{array}{l} \;\\ 0,0\\ 0,1\\ 1,0 \end{array} & \begin{array}{c} 0\;1\\ \left(\begin{array}{cc} 1 & 0\\ 0 & 1\\ 1 & 0 \end{array}\right). \end{array} \end{array}$$

In this instance, $I\;\left[X_{-1}:Y_{-1}\right]=0.449821$ bit while $I\;\left[X_{-1}:Y_{-1}|X_{0}\right]=0.0$ bit. This indicates that the variables form a Markov chain $X_{-1}-X_{0}-Y_{-1}$ and so any information $X_{-1}$ and $Y_{-1}$ share is contained within $X_{0}$. Combined with the synergistic flow being zero, the shared information flow is $I\;\left[X_{-1}:Y_{-1}:X_{0}\right]=0.449821$. From this, the intrinsic information flow from *Y* to *X* is $I\;\left[Y_{-1}:X_{0}\right]-0.449821\;\mathrm{bit}\;=\;0.526200\mathrm{bit}$.

Looking toward the *Y* time series, we find the following transition matrix:

(7) T[X−1,Y−1→Y0]= 0,00,11,00101104959.

Here, consider a locally-modified $Y_{-1}^{\prime }$ constructed by passing $Y_{-1}$ through a channel that preserves the value 0, but maps a 1 to a 0 with probability 1645. This results in the modified transition matrix:

(8) T[X−1,Y−1′→Y0]=0,00,11,0014959104959.

given $Y_{-1}^{\prime }$, $X_{-1}$, and $Y_{0}$ are independent.

This implies that no information about $Y_{0}$ can be uniquely attributed to $X_{-1}$, since there is a method of reconstructing any influence $X_{-1}$ has on $Y_{0}$ using $Y_{-1}$ alone. We then conclude that the intrinsic information flow from *X* to *Y* is 0.0 bit. Shared information flow is then $I\;\left[X_{-1}:Y_{0}\right]=0.044381$ bit, while synergistic flow is $I\;\left[X_{-1}:Y_{0}|Y_{-1}\right]=0.120759$ bit.

**Figure 5:**
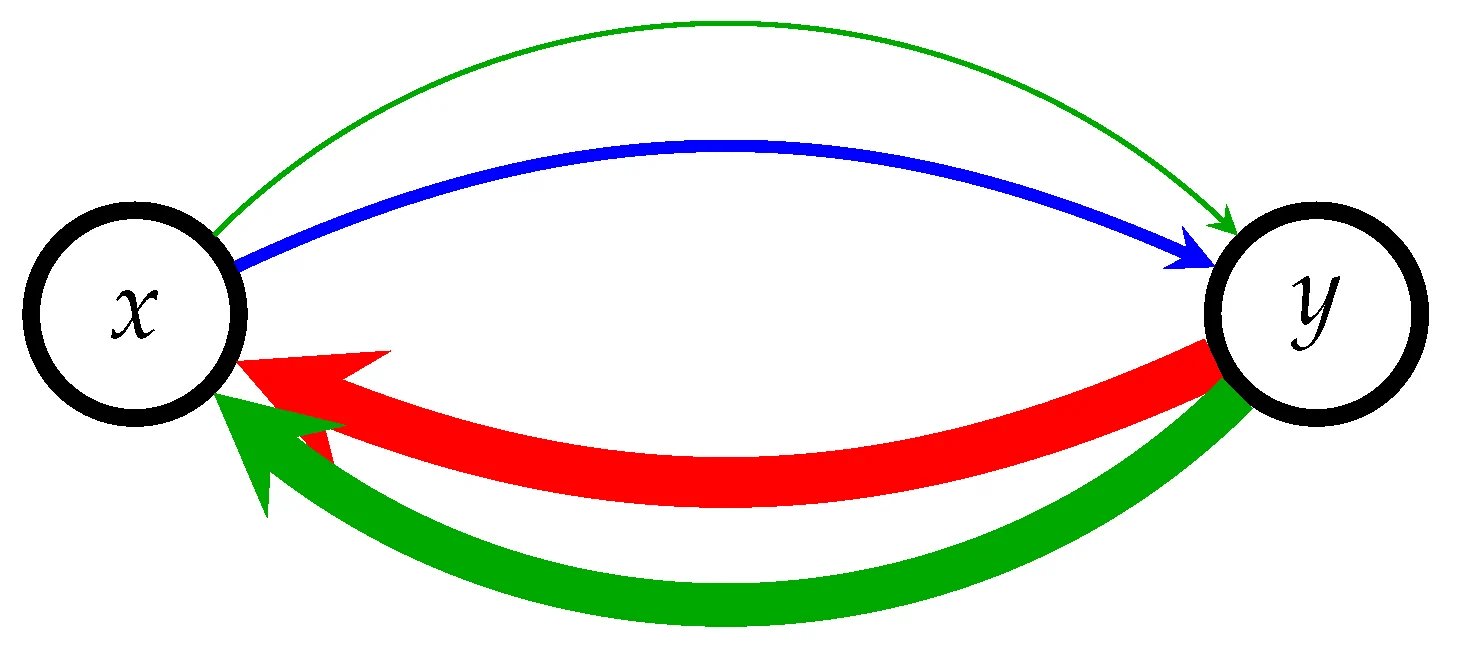
Information flows between *x* and *y*: The arrow color corresponds to flow mode: Red for intrinsic, green for shared, and blue for synergistic. Arrow width corresponds to the flow strength: Wider the arrow, the larger that flow mode. For the case considered in this section, there is relatively little flow from *x* to *y* and its modes are shared and synergistic, while there is significantly more information flow from *y* to *x* and its modes are intrinsic and shared.

These jointly Markovian time series exemplify the degree of asymmetry that can exist in information flow. Specifically, it is not immediately obvious that shared information flow—due to common driving or synchronization, for example—can be large in one direction while small or nonexistent in the other. These sorts of asymmetries appear in a variety of data sets, and so its demonstration in a relatively simple Markovian setting is pedagogically helpful and must be kept in mind when structurally interpreting the estimated flows. These relationships are summarized in Figure 5.

### 5.2. Coupled Chaotic Maps

Shifting from the setting of joint stochastic processes, consider now a pair of coupled deterministic, but chaotic dynamical systems—coupled tent maps of the unit square. Individually, a tent map is a simple discrete-time dynamical system that exhibits chaotic behavior. The update rule is given by:

$$\begin{array}{cc} x_{t+1} & =f(a,x_{t})\\ f(a,x) & =a\left(\frac{1}{2}-\left|x-\frac{1}{2}\right|\right)\;, \end{array}$$

where the initial condition $x_{0}\in \left[0,1\right]$. A tent map is chaotic for 1<a≤2. An *orbit* of the map—a temporal sequence $x_{0},x_{1},\ldots$—can be discretized via the mapping:

$$\begin{array}{c} s_{t}=\left\{\begin{array}{cc} 0 & \mathrm{if}\;x_{t}<\frac{1}{2}\\ 1 & \mathrm{otherwise} \end{array}\right.\;. \end{array}$$

This coarse binary discretization—mapping from a real number to a binary one—is known as a *generating partition* of the unit interval. Importantly, the resulting binary time series $s_{0},s_{1},\ldots$—preserves many dynamical properties of the continuous-space orbit, such as the degree of amplifying small variations in the state, captured by the Kolmogorov-Sinai entropy rate [32].

**Figure 6:**
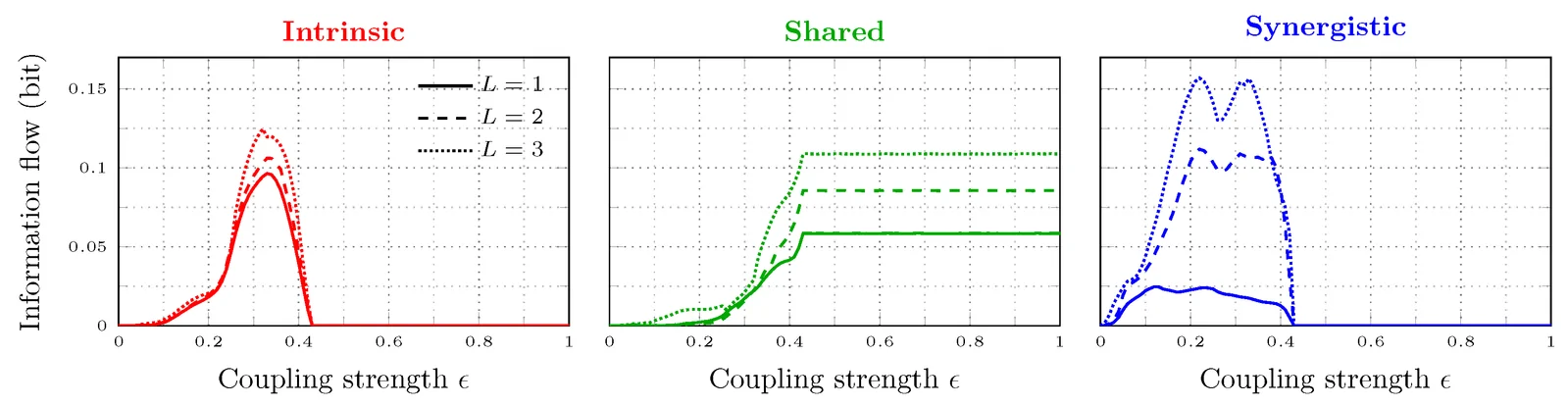
**Intrinsic**, **shared**, and **synergistic** information flows from *A* to *B* for the coupled tent maps with $a_{1}=a_{2}=1.75$, as a function of the coupling strength ϵ, for history lengths L=1 (solid), 2 (dashed), and 3 (dotted). Beyond ϵ≈0.43 the two maps synchronize, the entire flow is shared, and the time series from each map are the same.

We couple two such systems together via the mapping:

$$\begin{array}{cc} x_{t+1} & =f(a_{1},x_{t})\\ y_{t+1} & =f(a_{2},\epsilon x_{t}+\left(1-\epsilon \right)y_{t})\;, \end{array}$$

where ϵ∈[0,1] is a coupling strength. Furthermore, we discretize the two orbits individually via the same mapping as above, resulting in the discrete-time binary sequences $A_{t}$ and $B_{t}$. We can then estimate the intrinsic, shared, and synergistic information flows from the “source” map $x_{t}$ to the “target” map $y_{t}$, shown in Figure 6, from the sequence statistics in the time series $s_{0},s_{1},\ldots$ ($A_{t}$ and $B_{t}$ respectively) from each tent map.

The pasts are blocks of *L* symbols, $\left(A_{t-L},\ldots ,A_{t-1}\right)$ for the source and $\left(B_{t-L},\ldots ,B_{t-1}\right)$ for the target, while the present is $B_{t}$. To gauge the sensitivity of the decomposition to this choice, we report history lengths L=1, 2, and 3. Each estimate pools eight orbits of $2^{22}$ iterates, each following a transient of $10^{4}$ iterates. A uniform perturbation of magnitude $10^{-12}$ is added at every iterate, which prevents maps of slope 2 from collapsing onto the fixed point 0 in floating-point arithmetic.

**Figure 7:**
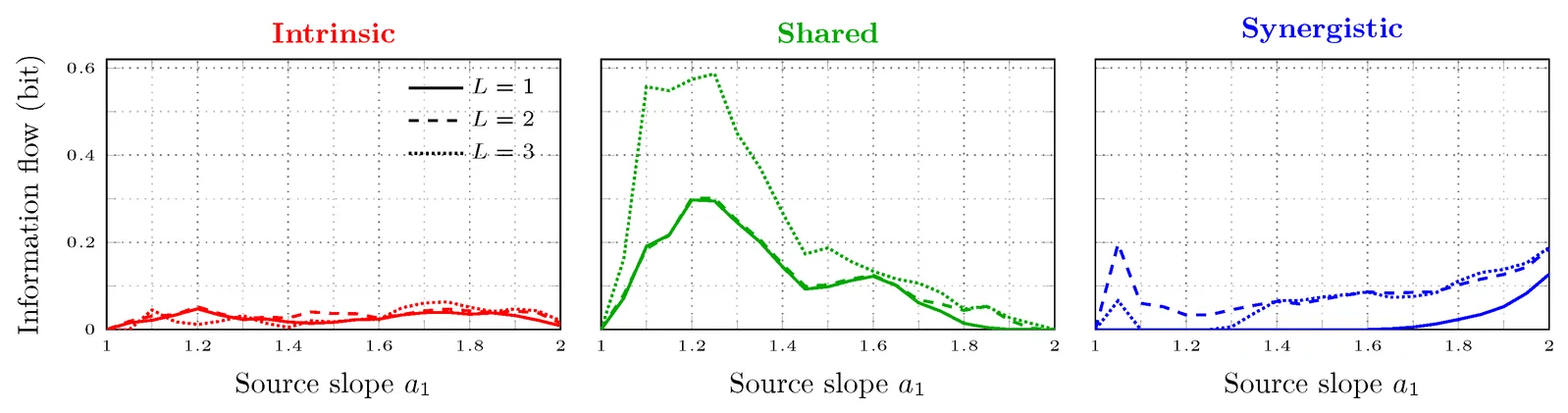
**Intrinsic**, **shared**, and **synergistic** information flows from *A* to *B* for the coupled tent maps at coupling ϵ=0.40 with the target slope fixed at $a_{2}=1.75$, as a function of the source slope $a_{1}$, for history lengths L=1 (solid), 2 (dashed), and 3 (dotted).

**Figure 8:**
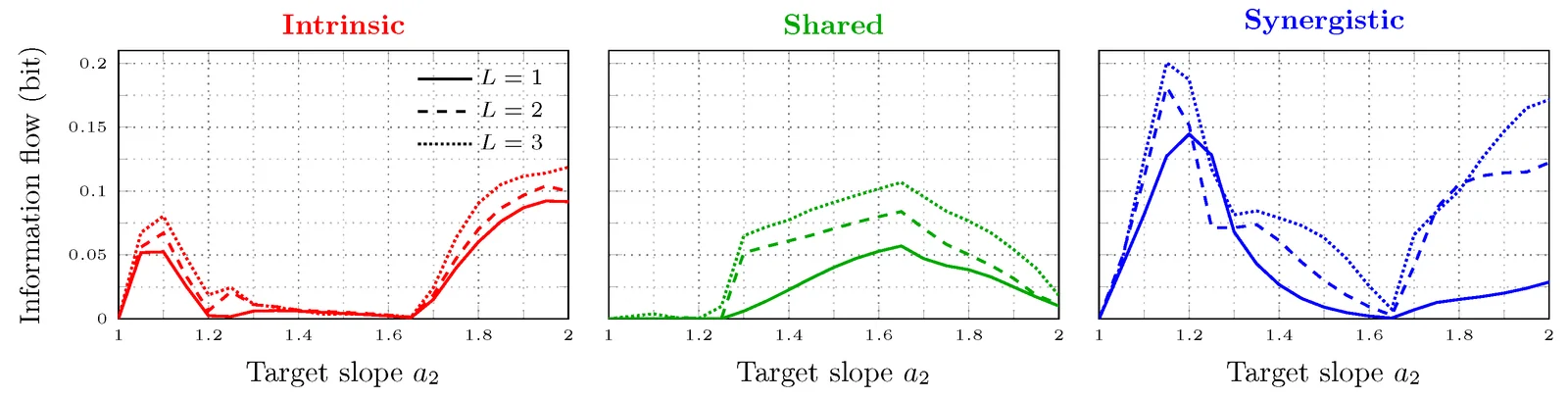
**Intrinsic**, **shared**, and **synergistic** information flows from *A* to *B* for the coupled tent maps at coupling ϵ=0.40 with the source slope fixed at $a_{1}=1.75$, as a function of the target slope $a_{2}$, for history lengths L=1 (solid), 2 (dashed), and 3 (dotted).

Alternatively, we can hold the coupling fixed at ϵ=0.40 and vary the slope of either map. Figure 7 varies the source slope $a_{1}$ while holding $a_{2}=1.75$, and Figure 8 varies the target slope $a_{2}$ while holding $a_{1}=1.75$.

Together these estimates of the informational modes demonstrate the range of possible information flows between the coupled chaotic dynamical systems.

In particular, Figure 6 demonstrates that for strongly coupled maps—above coupling ϵ≈0.43—the intrinsic and synergistic informations flows vanish and the shared information is maximized. This occurs since the states of the component maps become synchronized and so the binary time series from each is the same—all the available (chaotically generated) information is shared, with none arising from intrinsic or synergistic interactions.

This holds at every history length. The value of the shared flow there grows with *L* ( 0.058 bit, 0.086 bit, and 0.109 bit), since a longer block of the source’s past better predicts the next symbol of the synchronized pair.

Below that coupling strength one finds a rich variation in the maps’ dynamic behaviors. There is a peak in intrinsic flow around ϵ≈0.33, at the same location and of similar size (0.096 bit to 0.125 bit) for all three history lengths. Since both slopes are fixed, the individual maps are no more or less chaotic at this coupling; the peak instead reflects the driven map’s response to its driver. As ϵ grows from zero, the driver’s state increasingly determines the target’s next symbol, raising the intrinsic flow. As synchronization approaches, however, the target’s own past increasingly duplicates the driver’s, so that the driver’s influence becomes redundant with what the target’s past already reveals. The fraction of time steps at which the two symbols disagree falls from about 0.46 at weak coupling to 0.22 at ϵ=0.33 and to zero at ϵ≈0.43. The intrinsic flow thus peaks between the two regimes, where the target is strongly driven but not yet synchronized, while the shared flow rises toward its synchronized value. For ϵ∈(0,0.43) there is also a cooperative interaction between the maps’ dynamics that creates synergistic flow. Its magnitude, however, depends strongly on the history length. With L=1 it is modest, at most 0.025. With L=2 and L=3 it reaches 0.11 bit and 0.16 bit, and exceeds the intrinsic flow over most of the range. A single symbol of the target’s past is a coarse proxy for the target’s state. Longer histories resolve that state more finely, and conditioning on it then uncovers more of the source’s influence, consistent with the masking mechanism of Section 4.4.

In contrast, Figure 7 varies the nonlinearity parameter $a_{1}$ of the source, which sets its dynamical instability. For $a_{1}≲1.6$ the flow is predominantly shared: the source’s dynamics dominate the system and the target follows them, with a small intrinsic flow throughout. Whether this strong driving suppresses synergistic flow depends on the history length. With L=1 synergistic flow vanishes below $a_{1}\approx 1.75$, but it is present from $a_{1}\approx 1.15$ with L=2 and from $a_{1}\approx 1.35$ with L=3. At every history length it rises toward $a_{1}=2$, reaching 0.13 bit to 0.19 bit. For $a_{1}≲1.4$, the shared flow at L=3 is roughly double that at L=1 and L=2. This coincides with $a_{1}<\sqrt{2}$, where the source’s attractor splits into bands, and so its symbol sequence carries a periodic phase that, plausibly, only the longer block resolves. At $a_{1}=1.05$ the estimates depend on the initial conditions.

Finally, Figure 8 reveals a large peak in cooperative—that is synergistic—information flow when the second map is only moderately unstable, at $a_{2}\approx 1.15$–1.2 (0.15 bit to 0.20 bit), where the intrinsic and shared flows are small. This peak is robust to the history length. Again, the informational interpretation is that the two maps together are creating and communicating information. The shared flow is maximized at $a_{2}\approx 1.65$ for all three history lengths, where the intrinsic and synergistic flows nearly vanish. There $\left(1-\epsilon \right)a_{2}\approx 1$: the target is marginally contracting, with a slope close to the source’s, and the two symbol sequences nearly coincide. Above $a_{2}\approx 1.7$ the target is expanding. Intrinsic flow then dominates at L=1, while at L≥2 the synergistic flow is comparable to or larger than it.

Overall, one concludes that distinct information flows between coupled chaotic systems exhibit a range of different kinds of information flow, dominated at one or another nonlinear parameter and coupling strength by intrinsic, shared, and synergistic flows. Across all three sweeps, the locations of these features and the size of the intrinsic flow are robust to the history length. The magnitude of the synergistic flow is not. Comparisons of synergistic flow should therefore be made at a fixed, reported history length.

As a final note, the informational estimates here are empirical. However, we would like to point out for the case of nonlinear dynamical systems with good measurements (obtained via their generating partitions) that information measures such as those introduced here for flows can be analyzed exactly. Examples of the analytical methods we have in mind are given in Ref. [33].

### 5.3. Leader-Follower Dynamics

Following the 2018 preprint of this work, the framework put forth here has also been applied to the study of flocking behavior in bacteria [14]. There, through extensive use in a number of different flock behaviors, it was found that the intrinsic information flow better distinguishes the roles of leaders and followers in collective motion. A variety of information flow types, similar to those just seen, appear and govern to a large extent the cooperative and competitive group behaviors.

### 5.4. Financial Information Flows

We next analyze information flows between a financial index and its stocks. The value of a financial index is the weighted average of the value of its constituent stocks. Here, we highlight our analysis of the Standard & Poor’s 500 (S&P 500), while Appendix E compares information flows in the S&P 400 and S&P 600 indices. The S&P 500 consists of 500 “large cap” stocks, whose total value is approximately $23.9 trillion dollars or 80% of the US market. The other indices are smaller, engineered to reflect financial dynamics at other economic scales. The time series consist of whether the daily closing price of each stock and of the index rose (1) or did not rise (0) between 1 January 2000 and 31 December 2008, the period analyzed in Ref. [34]. We only include the 252 stocks whose symbol was in the index for the entirety of the date range and for which a complete price history is available. Index membership from https://github.com/fja05680/sp500, accessed on 22 September 2026; prices from Yahoo Finance and, for since-delisted stocks https://www.tiingo.com, accessed on 22 September 2026. We estimate each information flow measure utilizing a past of length 1. These methods closely follow those of Ref. [34], where the transfer entropy between the S&P 500 and its constituents was analyzed. We use the same window, symbolization, and history length as that study, so that our decomposition of the flows can be compared directly with its transfer entropy results. Furthermore, it is not lost on the authors that the various stock prices were not stationary over this time frame, nor would they be over any time frame long enough to reasonably gather statistics. We chose to ignore this here in the interest of demonstrating how our framework can be applied to empirical data. We do note however that splitting the data approximately in half and analyzing the two halves separately yields qualitatively similar results with regards to stock/index asymmetry.

The flows are small—most well below 0.005 bit—and plug-in estimates from N=2261 observations are biased upward by roughly 3 × 10^−4^ bit to 6 × 10^−4^ bit even between independent series. We therefore test each flow against surrogate data. Each mode vanishes under particular conditional independencies among $X_{-1}$, $Y_{0}$, and $Y_{-1}$: intrinsic flow whenever $I\;\left[X_{-1}\;:\;Y_{0}\;\;|\;\;Y_{-1}\right]$ or $I\;\left[X_{-1}\;:\;Y_{0}\right]$ does, synergistic flow whenever, e.g., $Y_{0}⊥\;⊥Y_{-1}|X_{-1}$. We realize each such condition by permuting one variable within strata of another. This preserves every other pairwise dependence, in particular the strong same-day co-movement of a stock and its index. (See Appendix F for details).

A mode is declared nonzero only if every condition under which it vanishes is rejected, an intersection–union test [35]. Since $I\;\left[X_{-1}\;:\;Y_{0}↓Y_{-1}\right]\leq \min\left(I\;\left[X_{-1}\;:\;Y_{0}\;\;|\;\;Y_{-1}\right],I\;\left[X_{-1}\;:\;Y_{0}\right]\right)$, the observed intrinsic flow is compared with the surrogate distributions of $I\;\left[X_{-1}\;:\;Y_{0}\;\;|\;\;Y_{-1}\right]$ and $I\;\left[X_{-1}\;:\;Y_{0}\right]$. Intrinsic flow is thus significant only when transfer entropy is—the hybrid approach of Section 5.5—which avoids the excess false positives of Ref. [16]. We use $10^{4}$ surrogates per test and control the false discovery rate at 5% within each mode and direction [36]. Claims about the population of stocks are assessed with a stationary block bootstrap over trading days [37], applied jointly to all series (mean block length 20 days, $10^{3}$ replicates). Details are in Appendix F.

**Figure 9:**
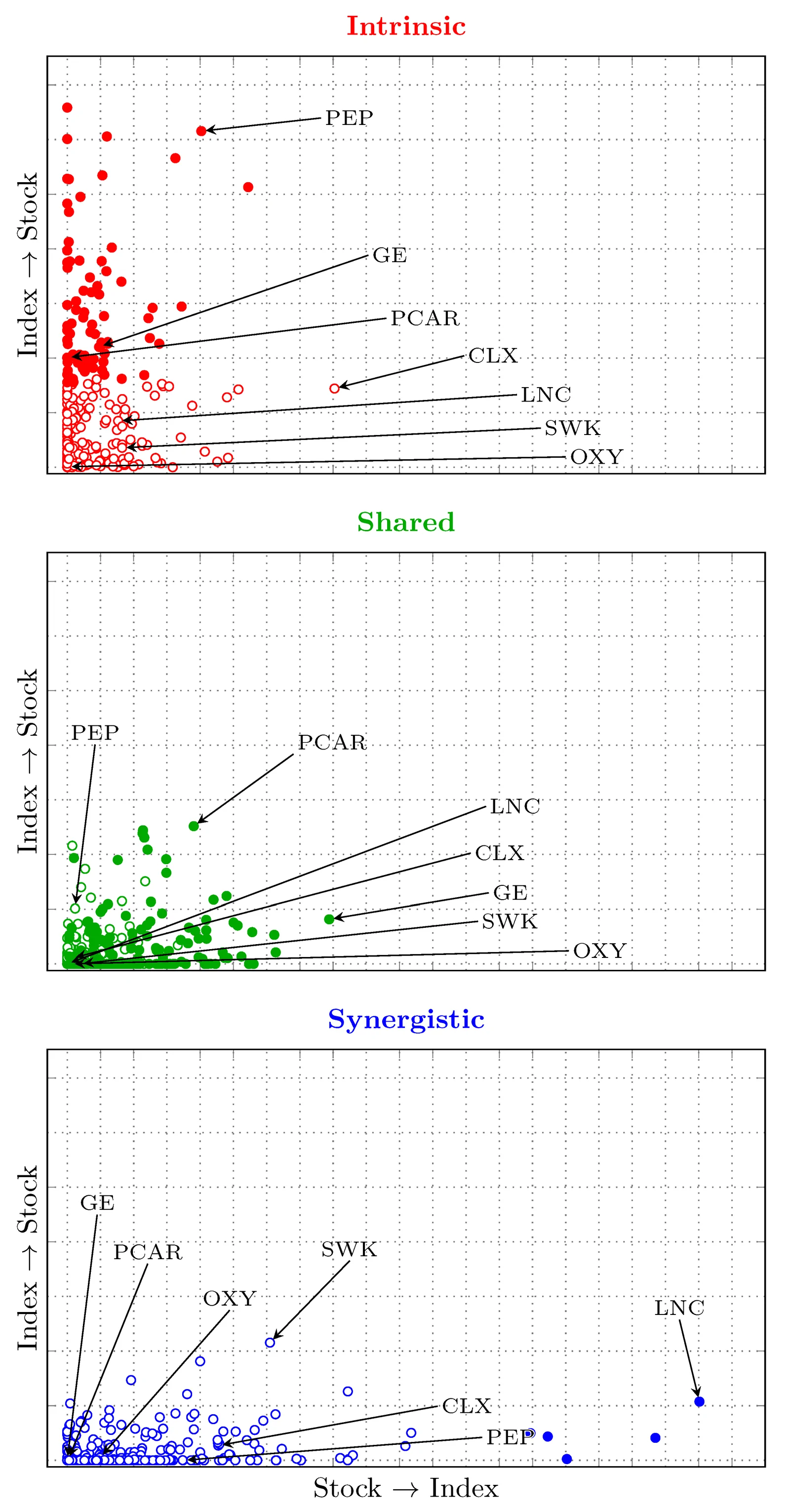
Distinct information flows within the “large cap” S&P 500 financial index. Each point is one of 252 stocks. Filled markers denote flows that are statistically significant (false discovery rate 5%) in at least one direction; hollow markers, in neither. No stock has a significant flow of the same mode in both directions. Axis scales are identical across the three panels: the horizontal axes run from 0.0000 bit to 0.0070 bit and the vertical axes from 0.0000 bit to 0.0126 bit.

To probe market behavior with our more refined scheme, we evaluate the intrinsic, shared, and synergistic information flows between each stock and its index. The S&P 500’s information flows are given in Figure 9. The analysis immediately reveals that intrinsic information flow is heavily skewed: the index intrinsically drives 80 of the 252 stocks, while no stock has a statistically significant intrinsic flow to the index. Across stocks, index-to-stock intrinsic flow exceeds stock-to-index by a median of $1.25\times 10^{-3}$ bit (95% confidence interval 0.39 × 10^−3^ to 2.34 × 10^−3^). Shared flow shows no significant aggregate asymmetry (median difference $-0.05\times 10^{-3}$ bit, interval −0.51 × 10^−3^ bit to 0.00 bit). Yet it is significant almost exclusively from stock to index: for 134 stocks, versus 2 in the opposite direction. This reflects the index’s own memory—its daily moves were mean-reverting over this period, $I\;\left[Y_{0}:Y_{-1}\right]=5.7\times 10^{-3}$ bit ($p\leq 10^{-4}$)—together with each stock’s same-day co-movement with the index.

A stock’s past is predictive of the index’s present through what it shares with the index’s past. Many of these shared flows are nonetheless small, so here significance reflects consistency rather than magnitude. Synergistic flow is skewed in the other direction (median difference $-0.29\times 10^{-3}$ bit, interval −0.71 × 10^−3^ bit to −0.02 × 10^−3^ bit): some joint feature of the prior values of the stock and index is predictive of the index value, but not so of the stock. Individually, however, synergistic flow is significant for only five stocks, all stock-to-index, so this asymmetry is a population-level effect assembled from many individually undetectable contributions. In Ref. [34], the asymmetry of the transfer entropy—the sum of intrinsic and synergistic flows—in the S&P 500 was noted. Our flow decomposition refines and strengthens this asymmetry. The excess of index-to-stock transfer entropy is carried by intrinsic flow and is detectable stock by stock. The smaller stock-to-index excess is synergistic and is detectable only in aggregate.

We can further analyze the behavior of specific stocks. PEP (PepsiCo), for example, is strongly driven—via intrinsic flow—by the behavior of the S&P 500, as are GE (General Electric) and PCAR (PACCAR Inc). Neither GE’s nor PCAR’s sizable shared flow from the index is significant. Instead, both have significant shared flow toward it; GE’s is the largest of any stock. LNC (Lincoln Financial Group) has the largest synergistic flow of any stock, and it runs from stock to index: LNC’s past behavior combined with that of the S&P 500 is predictive of the index. OXY (Occidental Petroleum) operates virtually independently from the S&P 500, with exceedingly little information flow of any mode in either direction. Finally, CLX (Clorox) has the largest stock-to-index intrinsic flow of any stock, yet it is not significant: apparent intrinsic influence of individual stocks on the index is consistent with estimation noise.

The S&P 400 and S&P 600 have also been analyzed, though without significance testing, and the results are in Appendix E. These mid- and small-cap stocks generally have less total information flow in both the stock-to-index and index-to-stock directions. Intrinsic flow is broader in these indices, without the pronounced asymmetry seen in the S&P 500. Shared flow, however, shows a strong asymmetry in the S&P 400 while is nonexistent in the S&P 600. Synergistic flow, again, shows none of the asymmetry that is seen in the S&P 500.

### 5.5. Calculation

Regarding the practical application of modes of information flow, two angles have been explored in works published after the 2018 preprint of this manuscript. Reference [15] explores a method of estimating the intrinsic transfer entropy in high-cardinality data. This methodology leverages large neural network estimators in order to avoid explicitly optimizing to find the mapping $Y_{-1}\to \bar{Y_{-1}}$. Reference [16] demonstrates that if one performs statistical hypothesis testing, for a fixed amount of data the null hypothesis is more likely to be erroneously rejected when using the intrinsic transfer entropy than using standard transfer entropy. This may limit the measure’s usefulness when inferring the presence or absence of intrinsic dependencies between systems, but does not imply that the quantification of that dependence is inappropriate. Rather, it suggests a hybrid approach: Use transfer entropy when testing for the presence of information flow, and use the intrinsic transfer entropy to quantify its strength. See also Appendix F for estimating statistical significance.

## 6. Conclusions

Detecting and quantifying information flow is both important and ill-defined—proposals to date have led to ambiguous, misleading, or inconsistent interpretations of behavior and structure. Conceptually, information flow is the medium through which causality propagates. Here, we proposed that one of the primary impediments to successfully diagnosing information flow is that it is not a singular concept. Rather, information flow can take on several qualitatively distinct modes. The intrinsic information flow is the mode most closely aligned with prior intuitions, such as that motivating the transfer entropy [20].

To quantify the intrinsic information flow, we proposed the cryptographic flow ansatz that posits intrinsic flow is synonymous with the ability to construct a secret key. This obviated the infinite regress of conditioning on all of a system’s other, possibly unspecified, components and the effects arising from conflating conditional independence and conditional dependence. This enabled us to quantify intrinsic information flow using the intrinsic mutual information, an easily computed upper bound on the secret key agreement rate. With this in hand, the remaining flow modes are quantified with the aid of the time delayed mutual information and the transfer entropy.

When appealing to cryptographic secret key agreement rate, we made a choice to approximate it with the intrinsic mutual information. Though, tighter upper bounds on the secret key agreement rate exist, they are generally much more difficult to estimate [38]. This makes them generally impractical in all but the smallest and simplest of cases. There also exist lower bounds on the secret key agreement rate [38], though these too are computationally prohibitive for general practice. Presumably, using improved bounds would be justified by an application’s need for more accuracy.

Refinements aside, the distinct quantification of each mode of information flow is broadly applicable. Demonstrating its consistency and discriminating power, we computed the intrinsic, shared, and synergistic information flows for key base cases and between several financial indices and their constituent stocks. As a new lens into stock market dynamics, these led to a significantly more nuanced view of the interactions between individual companies and the market. For example, we found that stocks whose transfer entropy from the S&P 500 is large are that way due to intrinsic flow, statistically significant for nearly a third of its constituents, while no stock detectably drives the S&P 500 intrinsically.

Additionally, shared information flow is often entirely neglected in analyses due to the prevailing opinion that transfer entropy supplants time-delayed mutual information whereas when considering information flow as multimodal the latter plays a first-class role. Without observations such as these it is impossible to paint a complete picture of how information is shuttled throughout a complex system. Looking forward, we believe that quantifying the distinct modes of information flow in an even broader variety of settings will lead to substantial improvements in our understanding of how a system and its components interact to generate truly complex behavior.

## Figures and Tables

**Figure A1:**
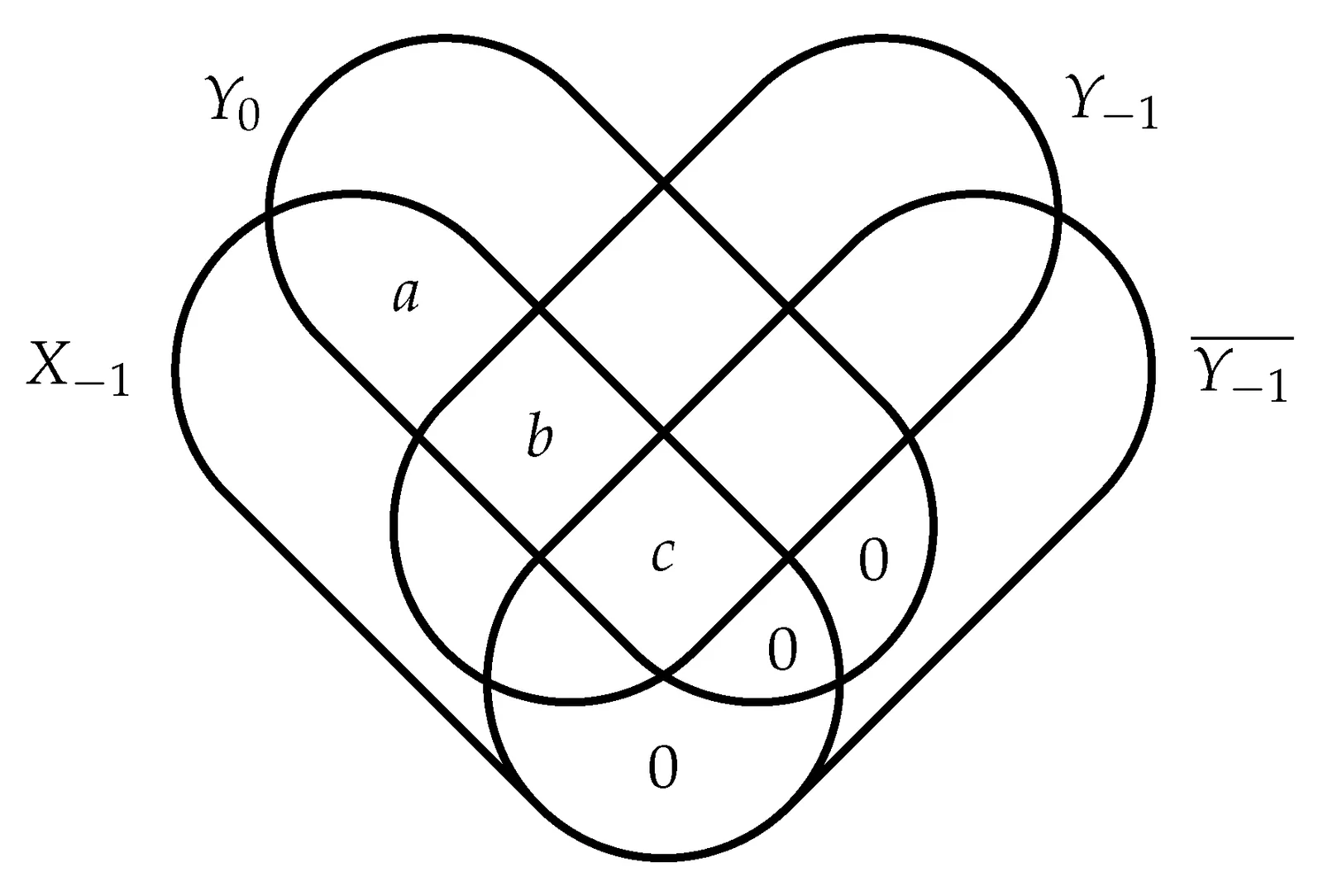
Analysis of how the three measures of information flow are related. From the definition of intrinsic mutual information, $I\;\left[X_{-1}Y_{0}:\bar{Y_{-1}}|Y_{-1}\right]=I\;\left[X_{-1}:\bar{Y_{-1}}|Y_{-1}\right]=I\;\left[Y_{0}:\bar{Y_{-1}}|Y_{-1}\right]=0$. Furthermore, b≤0. $I\;\left[X_{-1}\;:\;Y_{0}\right]=a+b+c$, $I\;\left[X_{-1}\;:\;Y_{0}\;\;|\;\;Y_{-1}\right]=a$, and $I\;\left[X_{-1}\;:\;Y_{0}↓Y_{-1}\right]=a+b$. From this, we can determine that intrinsic information flow is a+b, shared information flow is *c*, and synergistic flow is −b. Together, the total information flow is $a+c=I\;\left[X_{-1}:Y_{0}\right]-I\;\left[X_{-1}:Y_{0}:Y_{-1}|\bar{Y_{-1}}\right]$.

**Figure A2:**
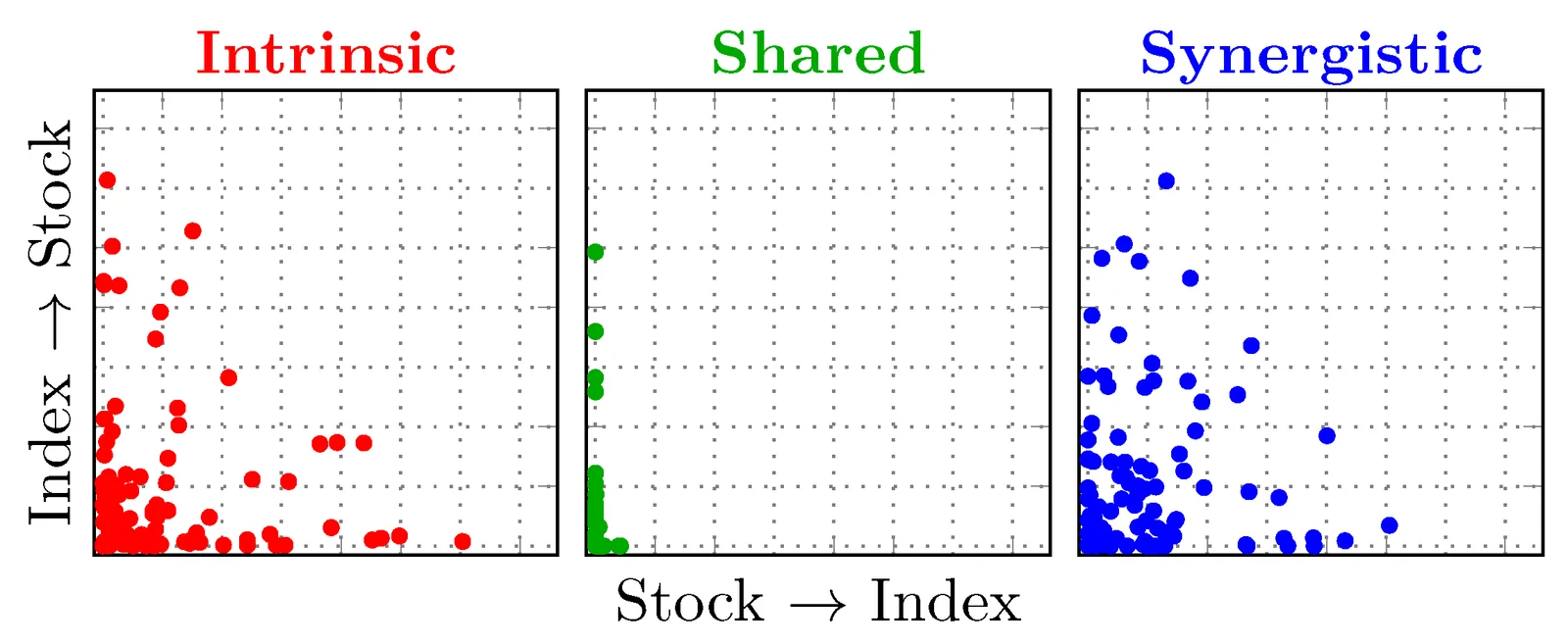
Information flows within the “mid cap” S&P 400. Axis scales are identical with minimum 0.0000 bit and maximum 0.0025 bit.

**Figure A3:**
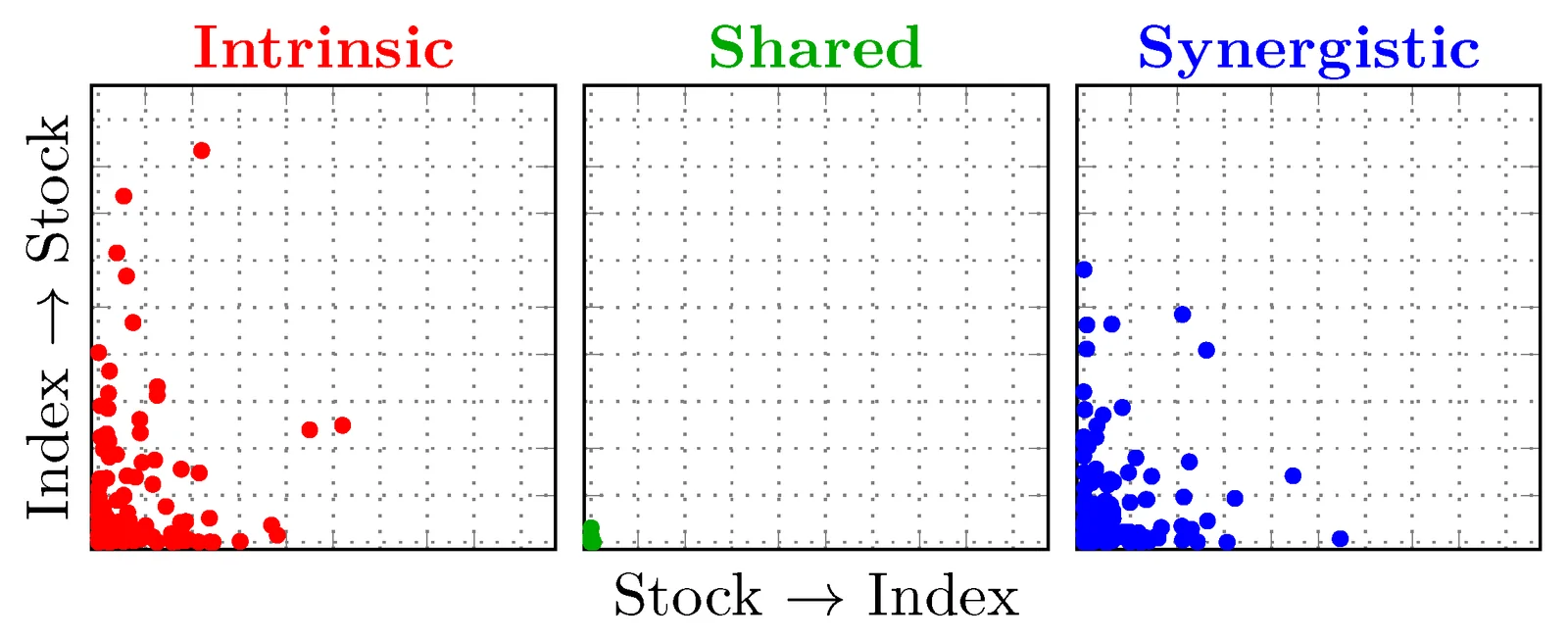
Information flows within the “small cap” S&P 600. Axis scales identical with minimum 0.0000 bit and maximum 0.0032 bit.

**Figure A4:**
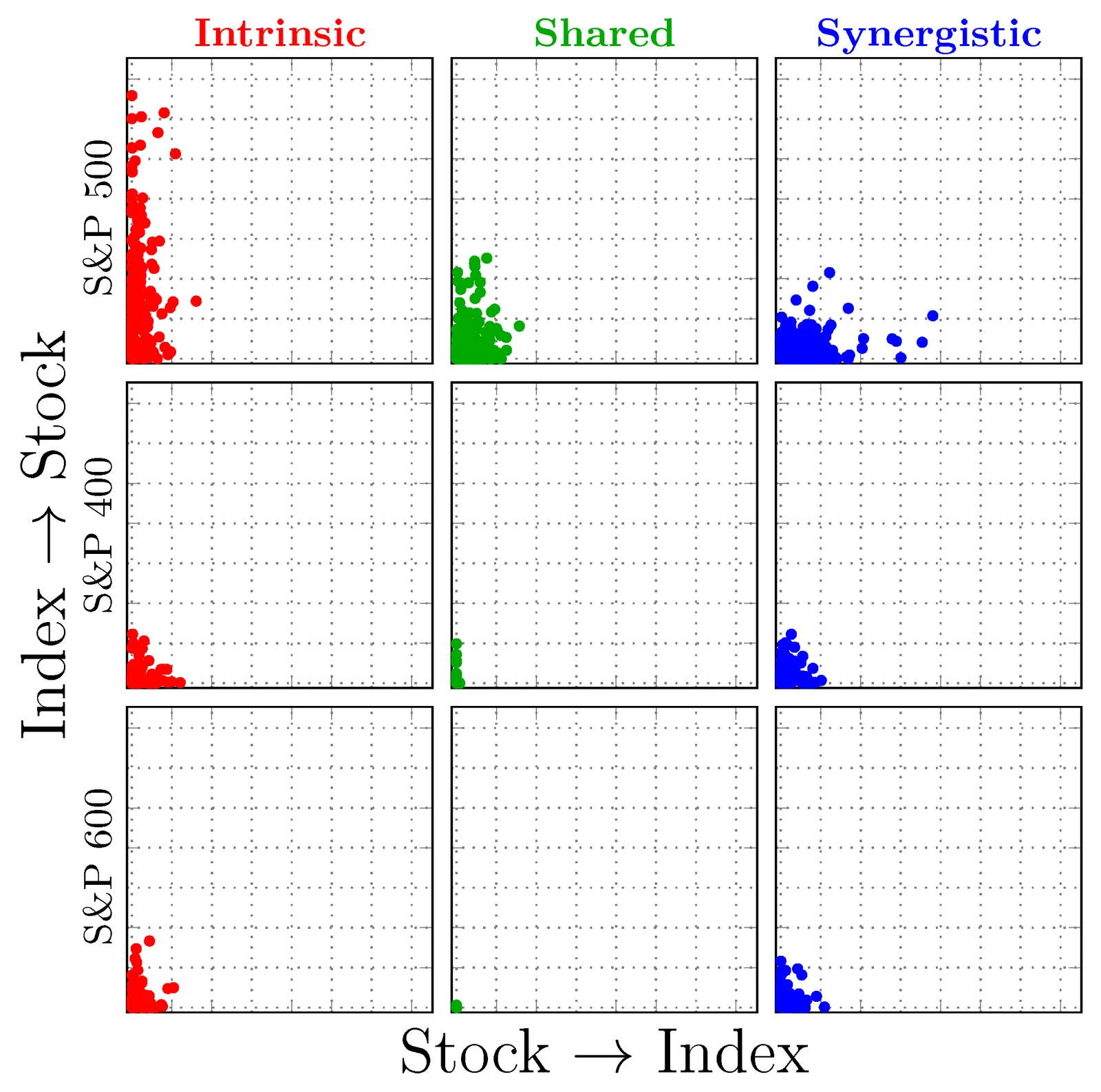
Information flows in the S&P 500, 400, and 600 stock indices compared. Axis scales identical with minimum 0.0000 bit and maximum 0.0126 bit.

## Data Availability

Data available upon reasonable request from the authors. Sources for empirical data are given within the text. Simulated data has the means of generation described explicitly within the text.

## Appendix A. Background: Times Series and Information

Let us summarize notation. We denote random variables using capital letters (*X*), realizations of random variables using lower case (*x*), and the event space of a random variable using calligraphics (X). We denote a sequence of temporally-ordered random variables (a time series) using a Python-like slice notation: $X_{t},X_{t+1},X_{t+2},\ldots ,X_{t+\tau -1}=X_{t:t+\tau }$. We suppress the starting or ending index of a slice if it is infinite; to wit, a bi-infinite time series is simply $X_{:}$. Throughout we assume stationarity—$X_{t:t+\tau }=X_{0:\tau }$—and that time series are ergodic. A time series is IID when it’s random variables are independent and identically distributed.

We next review several fundamental information-theoretic measures; for a more detailed introduction please refer to any standard text; e.g., Refs. [1,2,39]. The central measure of information theory is a random variable’s *entropy*:

(A1) $$\begin{array}{c} H\;\left[X\right]=-\sum_{x\in X}p\left(x\right)\log_{2}p(x)\;. \end{array}$$

The entropy of a joint variable is defined similarly:

(A2) $$\begin{array}{c} H\;\left[X,Y\right]=-\sum_{x,y\in X\times Y}p\left(x,y\right)\log_{2}p(x,y)\;. \end{array}$$

These quantify the total amount of uncertainty that exists within a set of random variables. Given two random variables, the *conditional entropy* quantifies uncertainty of one given knowledge of the other:

(A3) $$\begin{array}{c} H\;\left[X|Y\right]=H\;\left[X,Y\right]-H\;\left[Y\right]\;. \end{array}$$

That is, it is the uncertainty “left over” after the uncertainty of *Y* is removed from the joint uncertainty of *X* and *Y*.

Entropy is, generally, subadditive. Its degree of subadditivity is known as the *mutual information* and quantifies the dependence between two variables:

(A4) $$\begin{array}{cc} I\;\left[X:Y\right] & =H\;\left[Y\right]+H\;\left[X\right]-H\;\left[X,Y\right] \end{array}$$

(A5) $$\begin{array}{cc} \; & =H\;\left[X,Y\right]-H\;\left[X|Y\right]-H\;\left[Y|X\right] \end{array}$$

(A6) $$\begin{array}{cc} \; & =\sum_{x,y\in X,Y}p\left(x,y\right)\log_{2}\frac{p(x,y)}{p(x)p(y)}\;. \end{array}$$

The *conditional mutual information* measures the additional change in uncertainty about *Y* given *X*, when given a third variable *Z*:

(A7) $$\begin{array}{cc} I\;\left[X:Y|Z\right] & =I\;\left[Y|Z\right]-I\;\left[Y|X,Z\right] \end{array}$$

(A8) $$\begin{array}{cc} \; & =\sum_{x,y,z\in X,Y,Z}p\left(x,y|z\right)\log_{2}\frac{p(x,y|z)}{p(x|z)p(y|z)}\;. \end{array}$$

Note that conditioning can increase statistical dependence; that is, $I\;\left[X:Y|Z\right]>I\;\left[X:Y\right]$. This reflects the fact that conditional mutual information is sensitive to both intrinsic dependencies between *X* and *Y*, as well as dependencies induced by *Z*. In other words, dependence between *X* and *Y* may be revealed through their relationship with *Z*. Such dependencies can occur even when *X* and *Y* are independent: $I\;\left[X:Y\right]=0$.

References [40,41] review how these elementary information quantities extend to measure randomness and correlation in time series.

## Appendix B. Joint Analysis of Information Flow

To gain a greater understanding of how joint interactions among time series lead to the three modes of information flow, consider all variables of interest simultaneously. Figure A1 represents all interactions of $X_{-1}$, $X_{0}$, $Y_{-1}$, and $\bar{Y_{-1}}$ in the form of an *I-diagram* [42]. Three of the regions are identically zero due to the constraints placed on $\bar{Y_{-1}}$; namely, that the variables form a Markov chain $X_{-1}Y_{0}$—$Y_{-1}$—$\bar{Y_{-1}}$. The time delayed mutual information $I\;\left[X_{-1}\;:\;Y_{0}\right]=a+b+c$; the transfer entropy $I\;\left[X_{-1}\;:\;Y_{0}\;\;|\;\;Y_{-1}\right]=a$; and the intrinsic flow $I\;\left[X_{-1}\;:\;Y_{0}↓Y_{-1}\right]=a+b$. Since the intrinsic mutual information is bound from above by the conditional mutual information, we conclude that information atom *b* is necessarily nonpositive.

As noted, intrinsic flow is given by a+b. Shared flow is then given by the time delayed mutual information minus the intrinsic flow, which is *c*. Synergistic flow is given the transfer entropy minus the intrinsic flow, that is, −b. Together, these measure the total flow or a+c.

## Appendix C. Relationship with the Partial Information Decomposition

The separation of transfer entropy into two components is not a new idea. Utilizing the *partial information decomposition* (PID) [43] Williams et al. [44] decompose the transfer entropy from *X* to *Y* into two components: that *uniquely* provided by $X_{-1}$ (“state independent transfer”), and that *synergistically* provided by both $X_{-1}$ and $Y_{-1}$ (“state dependent transfer”):

(A9) $$\begin{array}{cc} I\;\left[X_{-1}\;:\;Y_{0}\;\;|\;\;Y_{-1}\right]= & I_{\cap }\left[X_{-1}\to Y_{0}\setminus Y_{-1}\right]+ \end{array}$$

(A10) $$\begin{array}{cc} \; & I_{\cap }\left[X_{-1}Y_{-1}\to Y_{0}\right]\;. \end{array}$$

While our intrinsic and synergistic information flows are qualitatively very similar to this idea, there are several important distinctions.

First, it is well-known that the intrinsic mutual information, used here to quantify intrinsic flow, is incompatible as a measure of unique information within the PID [45]. Our subsequent work bears this out. We proposed a measure of unique information based on how source–target dependencies constrain the joint distribution [46]. We also examined secret key agreement rates directly as candidate unique informations [47]. Of the four communication settings, only the one-way rate in which the target alone communicates yields a consistent PID; the two-way rate on which our cryptographic flow ansatz rests does not. Intrinsic flow is therefore not, and is not intended to be, a PID atom. Second, the PID’s goal is to decompose (for example) $I\;\left[X_{-1},Y_{-1}:Y_{0}\right]$, whereas our flows are not constrained to any particular sum (Section 4.3). Furthermore and finally, with the lack of a widely accepted method of computing the PID and the suggestion of its incompleteness when the number of inputs exceeds two [48], there is always the unfortunate possibility that the PID endeavor simply cannot be realized.

The closest neighbor of the present program is the integrated information decomposition (ΦID) of Mediano et al. [21]. For a system of two components *X* and *Y*, ΦID decomposes the time-delayed mutual information of the whole system, $I\;\left[X_{-1},Y_{-1}:X_{0},Y_{0}\right]$, over the product of two redundancy lattices: one for the components’ pasts and one for their presents. This yields sixteen atoms, each naming how information carried in a particular way in the past is carried in the future; for example, “redundant to synergistic”. Transfer entropy and time-delayed mutual information are both sums of these atoms, so ΦID resolves each of them more finely than our three modes do.

One sees, then, that the two programs differ in three ways. First, ΦID inherits the partial information decomposition’s dependence on a choice of measure: Shannon quantities fix only fifteen of the sixteen atoms, and the last requires a double-redundancy function, of which several have been proposed. Our decomposition instead rests on a single operationally-defined quantity, the secret key agreement rate, approximated by the intrinsic mutual information, and requires no redundancy measure. Second, ΦID partitions a fixed total, the system’s time-delayed mutual information, whereas our modes obey no such constraint (Section 4.3). Third, ΦID decomposes the joint dynamics of all components at once, whereas our flows are directed and pairwise. They are computed from $\left(X_{-1},Y_{0},Y_{-1}\right)$ alone and isolate the part of one component’s influence on another that no processing of the other’s own history can account for.

In short, the two approaches are therefore complementary. ΦID describes how information is carried by the system as a whole, while the cryptographic ansatz asks which part of a directed influence is private to the source and target.

## Appendix D. Computing the Intrinsic Mutual Information

All computations were performed with dit [49]. For discrete random variables, the corruption $\mathrm{Pr}(\bar{z}|z)$ is a row-stochastic matrix and, since it suffices to consider $|\bar{Z}\left|\;\leq \;|Z\right|$ [27], the minimization defining $I\;\left[X:Y↓Z\right]$ ranges over a compact, finite-dimensional set: a product of |Z| probability simplices, each of dimension |Z|−1. The objective $I\;\left[X:Y\;|\;\bar{Z}\right]$ is a continuous function of the matrix entries, so the minimum is attained. The objective need not be convex, however, and so dit searches for the global minimum using the basin hopping method from scipy.optimize. Basin hopping is not guaranteed to find the global minimum. To improve its chances, the implementation in dit combines several additional search strategies with a large number of restarts and iterations, and in controlled examples it reliably recovers the proven optima. One such strategy is to use the constant corruption and the identity corruption as two of the initial conditions seeding basin hopping. These yield $I\;\left[X:Y\right]$ and $I\;\left[X:Y\;|\;Z\right]$, respectively. In the flow setting they are the time-delayed mutual information and the transfer entropy, so the minima considered always include both, and the estimate never exceeds $\min\{I\;\left[X_{-1}\;:\;Y_{0}\right],I\;\left[X_{-1}\;:\;Y_{0}\;\;|\;\;Y_{-1}\right]\}$. It may nonetheless still stop at a local minimum. Since every corruption yields a value at or above the minimum, such a failure can only overestimate $I\;\left[X:Y↓Z\right]$. The reported intrinsic flows are therefore upper estimates of an upper bound on the secret key agreement rate.

### Appendix D.1. Other Bounds on the Secret Key Agreement Rate

The intrinsic mutual information is one of several known bounds on S(X:Y||Z). The simplest upper bound is $\min\{I\;\left[X:Y\right],I\;\left[X:Y\;|\;Z\right]\}$ [24], which the intrinsic mutual information improves upon. It can in turn be tightened. The *reduced* intrinsic mutual information [50], $\min_{U}I\;\left[X:Y↓ZU\right]+I\;\left[U\right]$, allows the eavesdropper to purchase additional side information *U* at a cost of $I\;\left[U\right]$. The *minimal* intrinsic mutual information [51,52], $\min_{U}I\;\left[X:Y\;|\;U\right]+I\;\left[XY:U\;|\;Z\right]$, is tighter still. The tightest known bound is the *two-part* intrinsic mutual information [51,52],

$$\begin{array}{cc} \underset{J}{\inf}\max_{V-U-XY-ZJ} & I\;\left[X:Y\;|\;J\right]\\ \; & +I\;\left[U:J\;|\;V\right]-I\;\left[U:Z\;|\;V\right]\;, \end{array}$$

where *J* is jointly distributed with (X,Y,Z). It splits the key into a part secret from *J* and a part that (X,Y) can conceal from *Z* but not from *J*. No cardinality bounds are known for its auxiliary variables, and so, unlike the others, it cannot be computed explicitly. Any particular choice of *J* nonetheless yields a valid upper bound [52]. A different tightening replaces the eavesdropper’s local corruptions with a weaker ordering of channels. Eve’s channel Pr(z|xy) is *less noisy* than a channel Pr(j|xy) if $I\;\left[U:Z\right]\geq I\;\left[U:J\right]$ for every U−XY−ZJ and every input distribution. In that case Alice and Bob can distill no more key against *Z* than against *J* [38]. Minimizing over all such *J* gives the *less-noisy* intrinsic mutual information [29]:

$$\begin{array}{c} I\;\left[{X:Y↓}_{\ln}Z\right]=\underset{J\;:\;\mathrm{Pr}\left(z|xy\right){⪰}_{\ln}\mathrm{Pr}\left(j|xy\right)}{\inf}I\;\left[X:Y\;|\;J\right]\;. \end{array}$$

Every corruption $\bar{Z}$ is dominated by *Z* in this sense, so it never exceeds $I\;\left[X:Y↓Z\right]$. It is never smaller than the two-part intrinsic mutual information, but it is not comparable in general to the reduced or minimal intrinsic mutual informations. It is the bound that certifies the vanishing key rate of the bound-information example of Ref. [29]. In the chain below, it may be placed between the intrinsic and the two-part intrinsic mutual informations. Together,

$$\begin{array}{cc} \min\{I\;\left[X:Y\right], & I\;\left[X:Y\;|\;Z\right]\}\\ \; & \geq I\;\left[X:Y↓Z\right]\\ \; & \geq \min_{U}I\;\left[X:Y↓ZU\right]+H\;\left[U\right]\\ \; & \geq \min_{U}I\;\left[X:Y\;|\;U\right]+I\;\left[XY:U\;|\;Z\right]\\ \; & \geq \underset{J}{\inf}\max_{V-U-XY-ZJ}I\;\left[X:Y\;|\;J\right]\\ \; & \;+I\;\left[U:J\;|\;V\right]-I\;\left[U:Z\;|\;V\right]\\ \; & \geq S(X\;:\;Y\;||\;Z)\;. \end{array}$$

From below, each restriction on the public communication yields a lower bound. Without any communication, Alice and Bob can agree only on their Gács–Körner common variable X⋏Y [53]. This is the part of *X* and *Y* that each can compute from their own observations. The key rate is then the conditional Gács–Körner common information K[*X* : *Y* | *Z*] [54]. When only Alice may speak, the rate is the one-way rate [55]:

$$\begin{array}{c} \max_{V-U-X-YZ}I\;\left[U:Y\;|\;V\right]-I\;\left[U:Z\;|\;V\right]\;. \end{array}$$

Taking *V* constant gives the secrecy capacity [56] $\max_{U-X-YZ}I\;\left[U:Y\right]-I\;\left[U:Z\right]$, the rate at which Alice could send Bob a secret over the channel X→(Y,Z). Choosing U=X in turn gives $I\;\left[X:Y\right]-I\;\left[X:Z\right]$ [24,55], and choosing U=X⋏Y recovers K[*X* : *Y* | *Z*]. Each of these has a mirror image with the roles of Alice and Bob exchanged. The larger of the two one-way rates is a lower bound on S(X:Y||Z)  [38]. Finally, allowing *k* rounds of alternating communication yields the interactive bound [51,57]:

$$\begin{array}{c} \max_{U_{0:k}}\max_{\zeta }\sum_{i\geq \zeta }I\;\left[U_{i}:W_{i}\;|\;U_{0:i}\right]-I\;\left[U_{i}:Z\;|\;U_{0:i}\right]\;. \end{array}$$

Here, Alice generates $U_{i}$ from $\left(X,U_{0:i}\right)$ on even rounds, with $W_{i}=Y$, and Bob generates it from $\left(Y,U_{0:i}\right)$ on odd rounds, with $W_{i}=X$. Maurer’s advantage distillation shows that interaction can be essential [24]: S(X:Y||Z) can be positive even when both one-way rates vanish. Together, the symmetrized versions of these bounds satisfy:

$$\begin{array}{cc} S(X\;:\;Y\;||\;Z) & \geq \mathrm{interactive}\\ \; & \geq \mathrm{one}\text{-}\mathrm{way}\\ \; & \geq \mathrm{secrecy}\;\mathrm{capacity}\\ \; & \geq \max\left\{\begin{array}{c} KX:Y\;|\;Z\\ I\;\left[X:Y\right]-I\;\left[X:Z\right]\\ I\;\left[X:Y\right]-I\;\left[Y:Z\right] \end{array}\right.\;. \end{array}$$

The intrinsic mutual information is not tight in general. Consider modifying the distribution of Figure 3 so that the eavesdropper observes Z=0 rather than 2 and Z=1 rather than 3. She can then no longer distinguish the 01-regime from the 23-regime. Here, $I\;\left[X:Y↓Z\right]=3/2$, while both the minimal intrinsic mutual information and the one-way lower bound equal 1 bit, so S(X:Y||Z)=1. The gap can even separate a positive bound from a vanishing rate. For the bound-information source of Ref. [29], $I\;\left[X:Y↓Z\right]$ is positive (approximately 0.001 bit), while the less-noisy intrinsic mutual information, and with it S(X:Y||Z), vanishes.

We nonetheless use the intrinsic mutual information because it strikes a balance between being nontrivial and being simple to compute. On the one hand, the simplest upper bound is too coarse for our purposes. Were intrinsic flow quantified by $\min\{I\;\left[X_{-1}\;:\;Y_{0}\right],I\;\left[X_{-1}\;:\;Y_{0}\;\;|\;\;Y_{-1}\right]\}$, then at least one of shared flow $I\;\left[X_{-1}\;:\;Y_{0}\right]-I\;\left[X_{-1}\;:\;Y_{0}↓Y_{-1}\right]$ and synergistic flow $I\;\left[X_{-1}\;:\;Y_{0}\;\;|\;\;Y_{-1}\right]-I\;\left[X_{-1}\;:\;Y_{0}↓Y_{-1}\right]$ would vanish identically, and the decomposition would be unable to detect both simultaneously. The intrinsic mutual information instead distinguishes conditional dependence from conditional independence, as Figure 3 demonstrates. On the other hand, the tighter bounds are considerably more expensive. The intrinsic mutual information optimizes over a single channel acting on *Z* alone, with $|\bar{Z}\left|\leq |Z\right|$, and so has ${\left|Z\right|}^{2}$ parameters. The reduced and minimal variants instead optimize over an auxiliary variable *U* that depends jointly on (X,Y,Z). With the cardinality bound |U|≤|X||Y||Z| used in dit, this requires ${\left(|X||Y||Z|\right)}^{2}$ parameters, or 64 rather than 4 even for binary series with pasts of length L=1. The lower bounds require maximizing over chains of auxiliary variables, whose cardinality bounds grow exponentially with the number of rounds [57]. Short of full interaction, they can also vanish when the rate is positive. Finally, every bound above lies below $\min\{I\;\left[X_{-1}\;:\;Y_{0}\right],I\;\left[X_{-1}\;:\;Y_{0}\;\;|\;\;Y_{-1}\right]\}$, so substituting any of them for $I\;\left[X_{-1}\;:\;Y_{0}↓Y_{-1}\right]$ keeps the decomposition nonnegative. A tighter bound would lower intrinsic flow and raise shared and synergistic flow by the same amount. Their difference, $I\;\left[X_{-1}\;:\;Y_{0}\right]-I\;\left[X_{-1}\;:\;Y_{0}\;\;|\;\;Y_{-1}\right]$, does not depend on which bound is used.

### Appendix D.2. Continuous Random Variables

While this calculation is straightforward for discrete probability distributions, there are significant difficulties in computing the intrinsic mutual information for continuous random variables. Barring a particularly clever and unforeseen method, these are:

1. *The search space is infinite dimensional.* When *Z* takes values in $R^{d}$, the corruption $\mathrm{Pr}(\bar{z}|z)$ is an arbitrary Markov kernel, and the cardinality bound no longer applies: $\bar{Z}$ may be discrete, continuous, or a mixture of the two. We know of no finite parametrization of the kernels guaranteed to contain a minimizer, nor is it guaranteed that the infimum is attained. In practice, one must establish a repertoire of potential transformations to apply to *Z*—quantizers, deterministic functions perturbed by additive noise, neural-network-parametrized kernels, and the like—and minimize over it. Any such restriction yields a value at or above $I\;\left[X:Y↓Z\right]$. This remains an upper bound on the secret key agreement rate, but there is no general way to certify how loose it is.
2. *Only information-destroying transformations help.* The conditional mutual information is invariant under invertible transformations of the conditioning variable: $I\;\left[X:Y\;|\;g(Z)\right]=I\;\left[X:Y\;|\;Z\right]$ for any bijection *g*. The useful corruptions are therefore precisely those that discard information about *Z*: many-to-one maps and added noise. The example of Figure 3 is typical: the optimal corruption merges outcomes of *Z*. Its continuous analog is a coarse-graining of *Z*, which produces an $\bar{Z}$ with discrete or mixed components.
3. *The objective must itself be estimated.* Outside of special parametric families, the density of (X,Y,Z) is unknown, and $I\;\left[X:Y\;|\;\bar{Z}\right]$ must be estimated from samples for every candidate corruption, e.g., with Ref. [58]’s nearest-neighbor estimator in its conditional form [59]. These estimators assume that a joint density exists. They are thus ill-suited to the discrete or mixed $\bar{Z}$ produced by exactly the corruptions identified in the previous item, since ties among samples make nearest-neighbor distances degenerate. Moreover, their bias depends on the dimension and distribution of $\bar{Z}$, and so varies from one candidate corruption to the next.
4. *Minimizing an estimate biases it downward.* The optimizer sees only a noisy estimate of the objective. Minimizing over many candidates preferentially selects those whose estimation error happens to be negative, since the expected minimum of estimates is no larger than the minimum of their expectations. The result can therefore underestimate $I\;\left[X:Y↓Z\right]$ even when each individual estimate is unbiased. Additionally, nearest-neighbor estimates are piecewise constant in the parameters of the corruption—they change only when the neighbor relations among samples change—so gradient-based optimization is unavailable, and each evaluation costs at least O(NlogN) for *N* samples.

The neural approach of Ref. [15], discussed in Section 5.5, is one way to address these difficulties. It trades the explicit search over corruptions for stochastic gradient training, but without guarantees of global optimality.

Parallel work treats secret key generation from Gaussian sources directly [60]. That machinery, however, cannot simply be plugged into the present framework, because discrete and differential information measures differ fundamentally. Differential entropies can be negative and are not invariant under changes of coordinates, and the Gaussian results rest on the special structure of jointly Gaussian sources. Neither property is available for the arbitrary continuous distributions, and the information-destroying corruptions of the second item above, that the intrinsic flow requires.

### Appendix D.3. Discretizing Continuous Signals

A practical alternative is to first discretize the continuous signals and then apply the discrete method described above. Both applications in Section 5 take this approach. Common discretization schemes include:

- *Binning*: partitioning the range of each signal into *k* intervals of either fixed width or equal probability. Equiprobable (quantile) bins maximize the entropy of the symbols and are insensitive to outliers and to monotone transformations of the signal.
- *Increments*: recording only the sign of each change, as in the financial application of Section 5.4.
- *Generating partitions*: when the dynamics are known, as for the tent maps of Section 5.2. The resulting symbol sequences preserve dynamical properties such as the Kolmogorov–Sinai entropy rate [32,61].
- *Ordinal patterns*: replacing each length-*m* window of the signal with the permutation that sorts it [62]. These are likewise invariant under monotone transformations.

Once discretized, the pasts and presents are formed as in the discrete case, the joint distribution of $\left(X_{-1},Y_{0},Y_{-1}\right)$ is estimated by counting, and $I\;\left[X_{-1}\;:\;Y_{0}↓Y_{-1}\right]$ is computed by optimizing over corruptions with $|\bar{Y_{-1}}\left|\;\leq \;\right|Y_{-1}|$.

Discretization affects the intrinsic mutual information in two opposing directions. Let *q* and *r* be the discretizations applied to *X* and *Y*, respectively. For any corruption, $I\;\left[q\left(X\right):r\left(Y\right)\;|\;\bar{Z}\right]\leq I\;\left[X:Y\;|\;\bar{Z}\right]$ by the data processing inequality [1]. Minimizing both sides over the same set of corruptions gives $I\;\left[q(X):r(Y)↓Z\right]\leq I\;\left[X:Y↓Z\right]$. Conversely, a discretization s(Z) of the eavesdropper’s variable is itself a corruption of *Z*, and every corruption of s(Z) is also a corruption of *Z*. Hence $I\;\left[X:Y↓s(Z)\right]\geq I\;\left[X:Y↓Z\right]$: coarse-graining can only weaken the eavesdropper. For $I\;\left[X_{-1}\;:\;Y_{0}↓Y_{-1}\right]$, the partition of *Y* applies both to $Y_{0}$ (a legitimate party) and to $Y_{-1}$ (the eavesdropper), so both effects occur, and the discretized value is in general neither an upper nor a lower bound on its continuous counterpart. We know of no result guaranteeing convergence under refinement of the partitions. This contrasts with mutual information, which is recovered in the limit of fine quantization [1]. A generating partition sidesteps this concern for deterministic dynamics. Otherwise, conclusions should be checked for robustness across a range of partitions.

Finally, finer partitions carry a statistical cost. With *k* symbols and pasts of length *L*, $Y_{-1}$ takes $k^{L}$ values, the joint distribution has $k^{2L+1}$ outcomes, and the corruption has $k^{2L}$ entries to optimize. Plug-in estimates of information quantities are biased by an amount that grows with the number of outcomes [63]. For independent variables, the plug-in conditional mutual information $I\;\left[X:Y\;|\;Z\right]$ is biased upward by approximately (|X|−1)(|Y|−1)|Z|/(2Nln2) bit, which for k=2, L=1, and N=2261 is the $6\times 10^{-4}$ bit observed in the financial application. Small alphabets and short pasts, together with the surrogate testing of Appendix F, are therefore advisable.

## Appendix E. Financial Indices Detailed Analysis

Here, we compare our analysis of the S&P 500 financial index in the main text to flows detected in the S&P 400 and S&P 600 indices. The S&P 400 consists of 400 “mid cap” stocks, whose total value is approximately $2.1 trillion dollars or 7% of the US market. The S&P 600 consists of 600 “small cap” stocks, whose total value is approximately $896 billion dollars or 3% of the US market.

Again, the time series consist of the sign of the change in the daily closing price of each stock and the index between 1 January 2000 and 31 December 2008 for stocks whose symbol was in the index for the entirety of the date range. And, as for the S&P 500, we estimate each information flow measure utilizing a past of length 1.

### Appendix E.1. S&P 400

The information flows within the S&P 400 are illustrated in Figure A2. These mid cap stocks display very different dynamics than the large cap S&P 500. Neither the intrinsic nor the synergistic flows display any marked asymmetry in directionality. The shared information flow, however, demonstrates a strong asymmetry where the stock and index are both predictive of the stock. Overall, the amount of information flow is significantly smaller than in the S&P 500.

### Appendix E.2. S&P 600

The S&P 600 dynamics are again different from the large and mid cap stocks. The intrinsic flows are skewed in a similar manner as the S&P 500, though not as strongly. Shared information flow, however, is effectively nonexistent. Synergistic flows are largely symmetric, similar to the mid cap stocks.

The dynamics of all three indices are displayed on equal scales in Figure A4.

## Appendix F. Statistical Significance of Financial Flows

The flow measures for financial time series are a concern as they are so low. The concern is compounded as the flow measures themselves are all nonnegative quantities and so empirical estimates are biased positive. And this increases the likelihood of specious positive estimates, when there are no flows.

So, to refine our tests of statistical significance of informational estimates we define *permuting one variable within strata of another* to mean holding the value of one variable fixed and shuffling others. This is a particularly helpful test for financial times series given our observation that the informational estimates are so low.

For example, to permute $X_{-1}$ within the *strata* of $Y_{-1}$, we take the entire time series of $X_{-1},Y_{-1},Y_{0},$ and identify the rows where $Y_{-1}=0$ (call this subset *A*) and where $Y_{-1}=1$ (call that subset *B*). Then, take all the $X_{-1}$ values in subset *A* and randomly permute them, keeping their distribution the same, but shuffling relative to $Y_{0}$. Then do the same within subset *B*. This makes $X_{-1}$ conditionally independent of $Y_{0}$ given Y_−1_. In this way, whatever the values estimated for the transfer entropy, intrinsic flow, or synergistic flow from *X* to *Y* they will be within the “noise” distribution since, in theory, the shuffling has rendered them independent. In short, whatever the estimated quantity—e.g., the transfer entropy—for the new partially permuted time series, that value is purely spurious.

With this mind, write $X=X_{-1}$, $Y=Y_{0}$, and $Z=Y_{-1}$. Table A1 lists four surrogate families. Each permutes one variable, globally or within strata of another, and so imposes exactly one conditional independence while preserving the remaining pairwise marginals.

**Table A1:** Surrogate families used to test each flow mode, with $X=X_{-1}$, $Y=Y_{0}$, and $Z=Y_{-1}$.

Permute	Imposes	Consequence
*X* within strata of *Z*	X⊥⊥Y|Z	$I\;\left[X:Y\;|\;Z\right]=I\;\left[X:Y↓Z\right]=0$, so synergistic flow =0
*X* globally	X⊥⊥(Y,Z)	$I\;\left[X:Y\right]=I\;\left[X:Y\;|\;Z\right]=0$
*Z* within strata of *X*	Y⊥⊥Z|X	synergistic flow =0, shared flow $=I\;\left[Y:Z\right]$
*Z* within strata of *Y*	X⊥⊥Z|Y	synergistic flow =0, shared flow $=I\;\left[X:Z\right]$

The last two rows follow from $I\;\left[X:Y\;|\;\bar{Z}\right]=I\;\left[X:Y\right]-I\;\left[Y:\bar{Z}\right]$ when Y⊥⊥Z|X, which is minimized by $\bar{Z}=Z$, and symmetrically for X⊥⊥Z|Y. For each mode, and each family *f* under which it vanishes, we form $p_{f}=\left(1+\#\left\{T_{b}\geq T_{\mathrm{obs}}\right\}\right)/\left(1+B\right)$ with $B=10^{4}$ surrogates, and report $p=\max_{f}p_{f}$. For synergistic flow, *T* is the synergistic flow itself under the first, third, and fourth families. For intrinsic flow, $T_{\mathrm{obs}}$ is the observed $I\;\left[X:Y↓Z\right]$ and $T_{b}$ is the surrogate $I\;\left[X:Y\;|\;Z\right]$ (first family) or $I\;\left[X:Y\right]$ (second family). This is valid since $I\;\left[X:Y↓Z\right]$ is bounded by both for every sample.

Shared flow vanishes when Y⊥⊥Z or X⊥⊥Z. The third and fourth families retain the dependence mediated by the remaining variable, so their surrogate shared flows are stochastically larger than under those conditions, and the test is conservative. Two limitations remain.

- The intrinsic test covers the sub-hypotheses $I\;\left[X:Y\;|\;Z\right]=0$ and $I\;\left[X:Y\right]=0$, but not distributions in which $I\;\left[X:Y↓Z\right]$ vanishes only through a nontrivial channel $Z\to \bar{Z}$.
- Permutation treats the overlapping observation triples as exchangeable.

The Markov chain of Section 5.1 probes the first limitation directly. Its flow from *X* to *Y* is exactly such a case. Both the transfer entropy ( 0.121 bit) and the time-delayed mutual information ( 0.044 bit) are positive, so neither sub-hypothesis holds, yet the intrinsic flow vanishes through a nontrivial channel on $Y_{-1}$. We simulated 1000 realizations of the chain at the financial sample size, N=2261, and applied the procedure above with B=2000 surrogates. In every realization the estimated transfer entropy and time-delayed mutual information were far above their surrogate nulls (at least 0.098 bit and 0.021 bit, respectively). The intrinsic flow from *X* to *Y*, however, was never declared nonzero at the 5% level: 0 of 1000 realizations, a 95% upper confidence bound on the false-detection rate of 0.3%. Meanwhile, the 0.526 bit intrinsic flow from *Y* to *X* was detected in all 1000. Here the estimated intrinsic flow is itself zero in every realization: the channel’s single free parameter can match the sample frequencies just as it matches the true ones, so the plug-in estimate inherits the exact zero. A distribution whose vanishing channel cannot be matched in-sample would instead yield small positive estimates, and remains untested by this example. Temporal dependence is addressed for the population-level claims by the stationary block bootstrap.
